# Metabolic adaptation of adherent-invasive *Escherichia coli* to exposure to bile salts

**DOI:** 10.1038/s41598-019-38628-1

**Published:** 2019-02-18

**Authors:** Julien Delmas, Lucie Gibold, Tiphanie Faïs, Sylvine Batista, Martin Leremboure, Clara Sinel, Emilie Vazeille, Vincent Cattoir, Anthony Buisson, Nicolas Barnich, Guillaume Dalmasso, Richard Bonnet

**Affiliations:** 1 Department of Bacteriology, University Hospital of Clermont-Ferrand, Clermont-Ferrand, France; 2 University Clermont Auvergne, Inserm U1071, INRA USC2018, M2iSH, F-63000 Clermont–Ferrand, France; 3 University Caen Normandie, équipe antibiorésistance EA, 4655 Caen, France; 4 University Clermont Auvergne, institut de Chimie, CNRS UMR 6296 Clermont-Ferrand, France; 5 Department of Hepato-Gastroenterology, University Hospital of Clermont-Ferrand, Clermont-Ferrand, France; 6 University Clermont Auvergne, Institut Universitaire de Technologie, Clermont-Ferrand, France

## Abstract

The adherent-invasive *Escherichia coli* (AIEC), which colonize the ileal mucosa of Crohn’s disease patients, adhere to intestinal epithelial cells, invade them and exacerbate intestinal inflammation. The high nutrient competition between the commensal microbiota and AIEC pathobiont requires the latter to occupy their own metabolic niches to survive and proliferate within the gut. In this study, a global RNA sequencing of AIEC strain LF82 has been used to observe the impact of bile salts on the expression of metabolic genes. The results showed a global up-regulation of genes involved in degradation and a down-regulation of those implicated in biosynthesis. The main up-regulated degradation pathways were ethanolamine, 1,2-propanediol and citrate utilization, as well as the methyl-citrate pathway. Our study reveals that ethanolamine utilization bestows a competitive advantage of AIEC strains that are metabolically capable of its degradation in the presence of bile salts. We observed that bile salts activated secondary metabolism pathways that communicate to provide an energy benefit to AIEC. Bile salts may be used by AIEC as an environmental signal to promote their colonization.

## Introduction

The adherent-invasive *Escherichia coli* (AIEC) pathogroup was initially characterized in isolates from the ileal mucosa of Crohn’s disease (CD) patients^1–5^. These bacteria strongly adhere to and invade intestinal epithelial cells (IECs), survive within macrophages, move into the deep tissues and activate immune cells to induce inflammatory cytokine secretion^6–8^. Several studies have identified genes that are important for AIEC ileal colonization: type 1 pili and flagella facilitate binding to and invasion of the epithelial cell; long polar fimbriae (LPF) aids in the binding of these bacteria to M cells overlying Peyer’s patches; Vat-AIEC protease promotes mucins degradation. The expression of most these virulence factors is modulated by bile salts, suggesting that bile salts may be used by AIEC as an environmental signal to promote their colonization^9–12^. Other genes identified to date as required for colonization of AIEC are involved in the acquisition and metabolism of essential nutrients^13^. In recent years, a huge body of literature has provided evidence that most enteropathogens are equipped with a large set of specific metabolic pathways to overcome nutritional limitations *in vivo*, thus increasing bacterial fitness during infection^14^. These adaptations include the degradation of myo-inositol, ethanolamine cleaved from phospholipids, fucose derived from mucosal glycoconjugates, 1,2-propanediol as the fermentation product of fucose or rhamnose and several other metabolites not accessible for commensal bacteria or present in competition-free microenvironments. Therefore, deciphering the metabolic requirements of AIEC is central to the understanding of their ability to colonize a host. In addition, little information is available regarding the effects of bile salts on *E*. *coli* metabolism. In the present study, we showed that secondary metabolic pathways in AIEC are modulated by bile salts, providing them with energy as well as carbon and nitrogen sources to colonize the intestinal mucosa.

## Results

### Bile salts altered expression of metabolic genes in LF82

For transcriptome analysis, an AIEC strain (LF82) isolated from a chronic ileal lesion of a patient with CD was grown for 24 h in the presence or absence of 1% bile salts^6^. The analysis of data showed a profound effect of bile salts with 1796 differentially expressed genes (DEG), including 1138 genes that showed an increase and 658 genes that showed a decrease in mRNA abundance, representing approximately 40% of the genome (additional file 1: Methods, Fig. S1, S2). Among these DEGs, 517 genes (29%) encoded proteins of unknown function.

**Figure 1:**
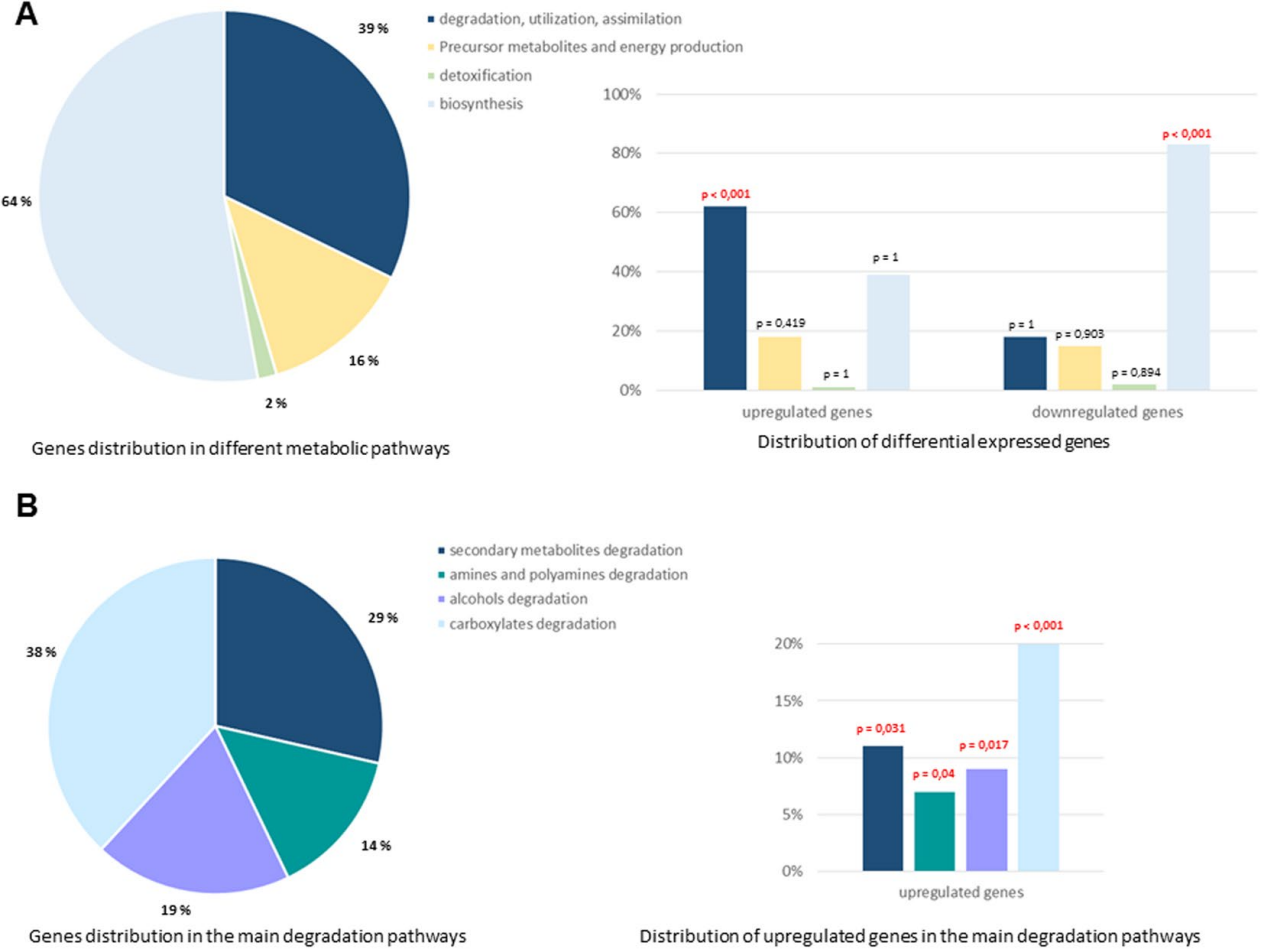
Bile salts induce a global up-regulation of LF82 genes involved in degradation pathways and a down-regulation of those implicated in biosynthetic pathways. Gene expression from AIEC strain LF82 after growth in either mM9 minimal medium or mM9 supplemented with bile salts. The proportion of up- and downregulated genes (upon bile salts exposure relative to control) in major biological pathways of metabolism was compared to those in the whole transcriptome background using a hypergeometric test. A p value ≤ 0.05 was considered as significant. (**A**) Distribution of the upregulated and the downregulated genes in the different metabolic pathways. The percentage of LF82 genes involved in every metabolic pathway is indicated in pie chart. The percentage of increased transcript levels and of decreased transcript levels in every pathway are represented on the right: 62% genes encoding proteins involved in the degradation, utilization and assimilation of compounds were up-regulated (p < 0,001) and 83% genes involved in biosynthesis pathways were down-regulated (p < 0,001) in the presence of bile salts. (**B**) Distribution of the upregulated genes in main degradation pathways. The percentage of LF82 genes involved in each pathway is indicated in pie chart. The percentage of increased transcript levels in every degradation pathways are presented on the right.

After classification into functional categories, we observed 255 DEGs among the 722 genes involved in metabolism (35%), including 182 up-regulated and 73 down-regulated genes. In the presence of bile salts, the expression of genes encoding proteins involved in the degradation, utilization and assimilation of compounds was significantly induced relative to the other overexpressed genes involved in metabolism (Fig. 1A). Conversely, bile salts induced a repression of genes involved in biosynthesis relative to the other underexpressed genes involved in metabolism. More particularly, the expression of genes involved in the degradation of amines, alcohols, carboxylates and in secondary metabolism were overexpressed for 47% of them (Fig. 1B). Thus, bile salts induce a global up-regulation of genes involved in degradation. We identified the most highly expressed genes, i.e., those showing an increase of at least 4-fold (i.e., log2 = 2) in mRNA abundance in bacteria grown with bile salts in comparison to bacteria grown without bile salts. These most up-regulated genes encoded proteins involved in sugar degradation (rhamnose, arabinose, galactose and mannose), L-ascorbate degradation, amino acid degradation (tryptophane, glutamine and arginine), citrate degradation, the methyl-citrate pathway and ethanolamine utilization. Ethanolamine (EA) can be used as a carbon and/or nitrogen source by many bacterial species and provide acetyl-CoA. Citrate degradation and the methyl citrate pathway provide acetate and pyruvate, respectively. Since acetyl-coA, acetate and pyruvate are central metabolic intermediates, we focused on these three pathways (additional file 1: Fig. S3).

### Induction of ethanolamine metabolism by bile salts

**Figure 2:**
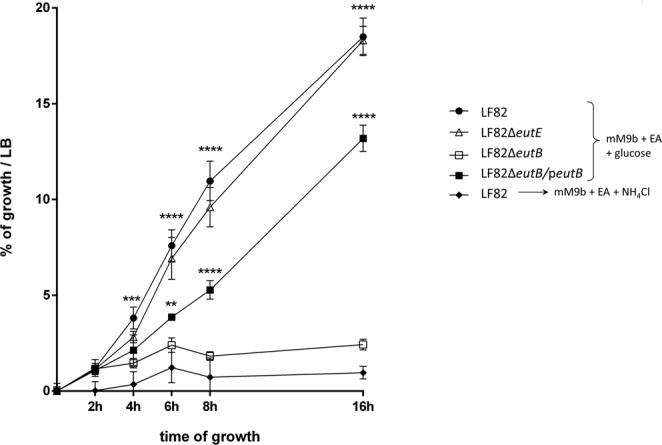
AIEC strain LF82 can use ethanolamine as a sole nitrogen source in presence of bile salts. All the strains were incubated at 37 °C with shaking in mM9 supplemented with bile salts (mM9b), ethanolamine (EA) and glucose or NH_4_Cl. LF82 was incubated in mM9b with EA as the sole source of carbon (filled rhombus) and mM9b with EA as the sole source of nitrogen (filled circles); LF82Δ*eutB* (open squares), LF82Δ*eutE* (open triangles) and LF82Δ*eutB*/p*eutB* (filled squares) were incubated in EA as the sole source of nitrogen. The growth of the strains was calculated as a percentage compared with their growth in LB medium for 16 h at 37 °C. No difference in growth was observed in LB medium between the LF82 strain and its mutants. Values are means ± SEM of at least three independent experiments (ANOVA; **p < 0.01; ***p < 0.001; ****p < 0.0001).

The genetic information required for the degradation and utilization of EA by *E*. *coli* is encoded by 17 genes in the *eut* operon. In the presence of bile salts, all of these genes were significantly up-regulated in AIEC LF82 (2.5 to 12.2-fold, Table 1). The intestinal tract provides a rich source of EA in the form of phosphatidylethanolamine due to its presence in bacterial and eukaryotic cell membranes and in the host diet. To evaluate the ability of the AIEC LF82 strain to utilize EA as a nitrogen source, EA was added along with 0.1% glucose to mM9 minimal medium supplemented with bile salts (mM9b). To test the ability of the bacterium to use EA as a carbon source, EA and NH_4_Cl were added to mM9b. As shown in Fig. 2, the AIEC LF82 strain failed to grow in minimal medium supplemented with EA and NH_4_Cl. This finding demonstrated that the LF82 strain is unable to utilize EA as a carbon source but can use EA as the sole nitrogen source. Ethanolamine ammonia lyase, encoded by the genes *eutB* and *eutC*, converts EA to ammonia and acetaldehyde (additional file 1: Fig. S3). While ammonia can serve as a cellular source of reduced nitrogen, acetaldehyde is further converted to acetyl-coenzyme A by an aldehyde oxidoreductase encoded by *eutE* and enters the carbon pool of the cell. As shown in Fig. 2, the mutant defective for EutB (LF82Δ*eutB*) was unable to grow in mM9b medium with EA as the sole nitrogen source, while complementation *in trans* with the *eutB* gene (LF82Δ*eutB/*p*eutB*) restored bacterial growth. In contrast, the LF82Δ*eutE* mutant showed a growth phenotype similar to the LF82 strain. These results clearly demonstrated that the AIEC strain LF82 can use EA as a nitrogen source and that a functional ethanolamine ammonia lyase including EutB is required for the release of ammonia.

**Table 1:** Variations of the expression of *eut* genes.

name	gene size	RNA-seq values (minimal medium)	RNA-seq values (bile salts)	Fold change	Fold change	p value
	(bp)	RPKM	RPKM		(Log_2_)	
*eutA*	1403	36	223	**6.3**	2.65	2.151E-13
*eutB*	1361	61	358	**5.9**	2.55	1.818E-17
*eutC*	887	47	286	**6.1**	2.60	5.747E-17
*eutD*	1016	108	357	**3.3**	1.72	2.746E-11
*eutE*	1403	14	149	**10.4**	3.38	1.849E-16
*eutG*	1187	35	168	**4.9**	2.28	2.691E-11
*eutH*	1226	106	514	**4.8**	2.27	1.172E-15
*eutJ*	836	13	147	**11.3**	3.50	8.290E-18
*eutK*	500	37	94	**2.5**	1.34	9.218E-05
*eutL*	659	30	126	**4.2**	2.08	1.825E-09
*eutM*	335	55	177	**3.2**	1.69	1.128E-05
*eutN*	287	5	62	**12.2**	3.61	1.249E-12
*eutP*	479	39	166	**4.2**	2.08	7.469E-10
*eutQ*	701	24	192	**8.1**	3.01	1.052E-12
*eutR*	1052	142	308	**2.2**	1.12	3.639E-05
*eutS*	335	20	76	**3.9**	1.96	8.018E-07
*eutT*	803	13	83	**6.4**	2.68	6.423E-10

^**1**^Values shown in bold are significant.

### Utilization of ethanolamine confers a competitive advantage to AIEC strains

**Figure 3:**
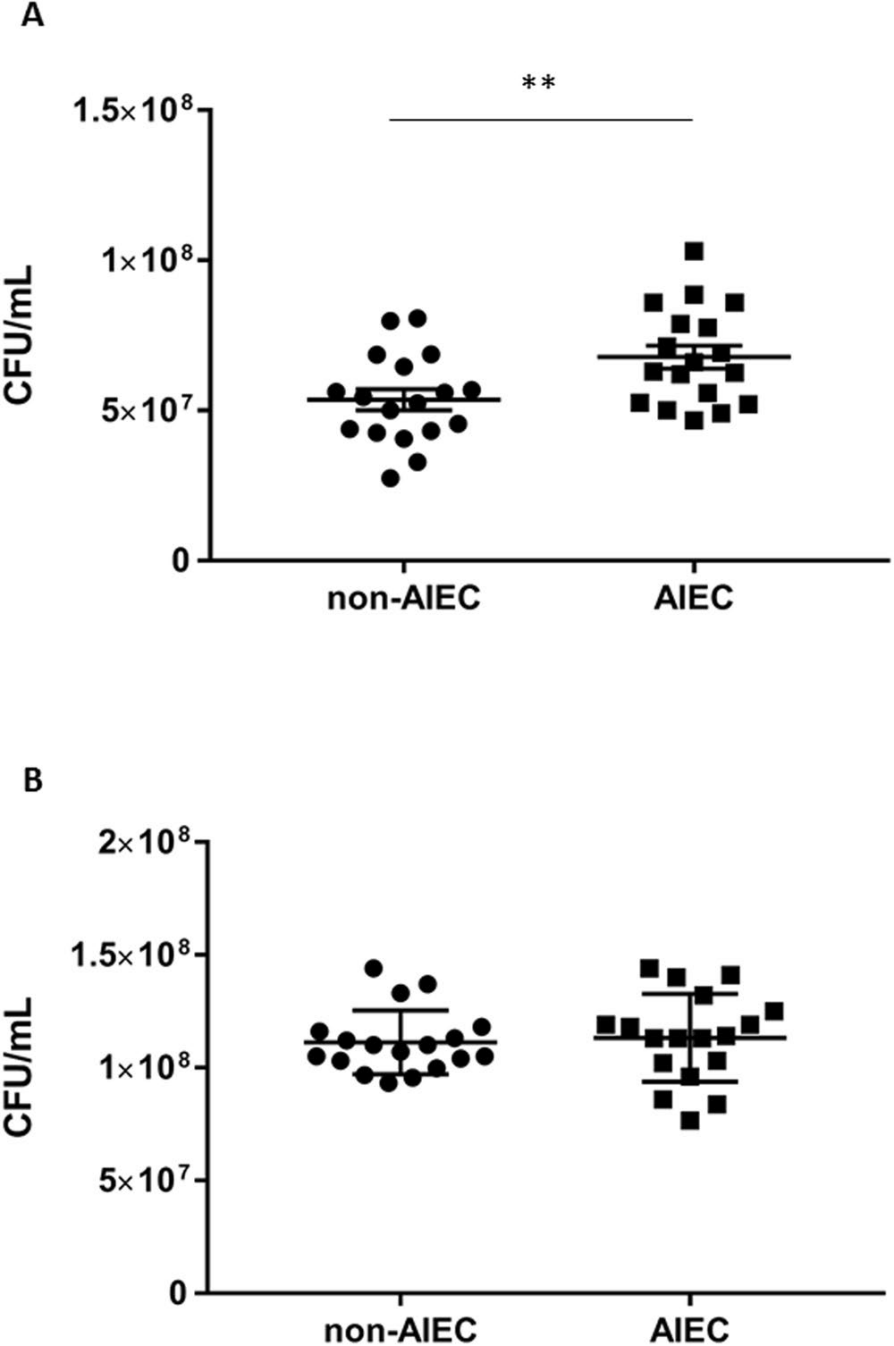
Comparison of ethanolamine utilization in AIEC and non-AIEC strains after 24 h of incubation in minimal medium. Data are presented as mean values ± SEM of endpoint culture from AIEC (n = 18) and non-AIEC strains (n = 18) isolated of ileal mucosa of CD patients. (**A**) Number of bacteria in mM9b supplemented with ethanolamine after incubation for 24 h at 37 °C. (**B**) Number of bacteria in mM9b supplemented with glucose and NH_4_Cl after incubation for 24 h at 37 °C. The growth of AIEC strains in mM9b-EA was slightly higher than that of non-AIEC strains. Values of each strains are means of two independent experiments. Statistical analysis was performed using a Student’s t test; **p < 0.01.

**Figure 4:**
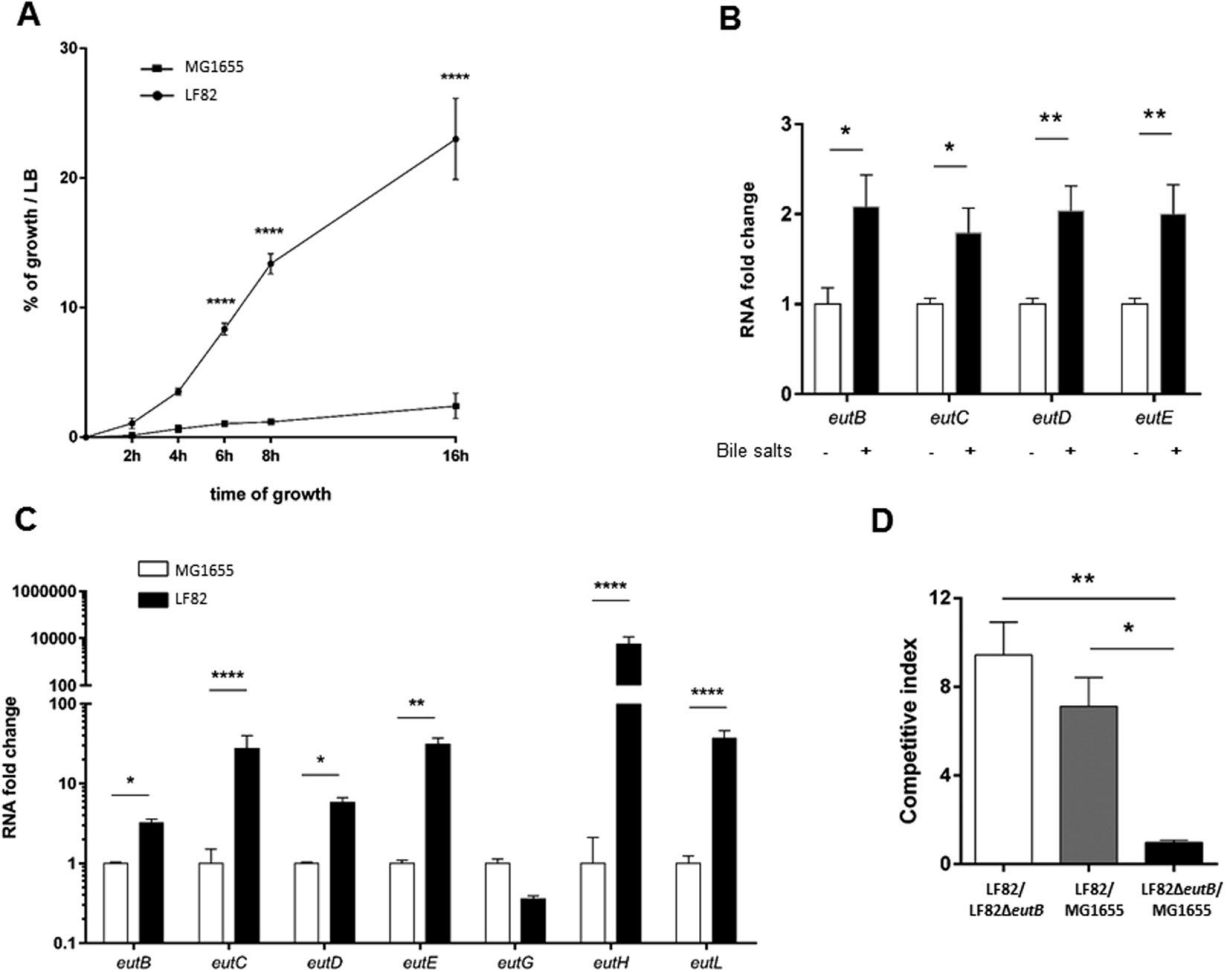
Ethanolamine utilization by AIEC strain LF82 provides a growth advantage against the avirulent *E*. *coli* MG1655 strain. (**A**) Bacterial growth curves of LF82 and MG1655 strains in mM9-EA supplemented with bile salts. The growth of strains in minimal medium was evaluated in comparison to the maximal of growth of each strain in LB medium (**B**) Relative expression levels of the *eut* genes in the LF82 strain. LF82 strains were grown at 37 °C to an OD600 of 0.6 in mM9-EA. Then, bile salts (1%) were added or not for 4 h. (**C**) Relative expression levels of the *eut* genes. The ratio of the mRNA level of each gene was measured in the LF82 strain and in the MG1655 strain after 8 hours of culture in the presence of bile salts. (**D**) Competitive index between LF82, LF82Δ*eutB* and/or MG1655 in mM9b-EA. Values are means ± SEM of at least three independent experiments. Statistical analysis was performed with the Mann-Whitney test or ANOVA test for multiple comparisons; *p < 0.05; **p < 0.01; ****p < 0.0001.

The *eut* operon is conserved within *E*. *coli* species. In order to determine whether the utilization of EA in presence of bile salts is a common trait shared by AIEC strains, we studied a collection of *Escherichia coli* isolates including AIEC (n = 18) and non-AIEC strains (n = 18). Non-AIEC strains grew more slowly in mM9b minimal medium supplemented with glucose (0.01%) and EA than AIEC strains (p = 0.009, Fig. 3A, additional file 1: Fig. S4). Nevertheless, growth was similar between group strains in mM9b minimal medium supplemented with glucose (0.1%) and NH_4_Cl (Fig. 3B, additional file 1: Fig. S4). Thus, ethanolamine metabolism could be a trait associated with AIEC strains. The growth of the AIEC LF82 strain was high in comparison to those of the *E*. *coli* strain MG1655 in mM9b-EA (Fig. 4A; additional file 1: Fig. S5). To explain the difference in growth, the ratio of the mRNA level of several genes of the *eut* operon was measured in both strains during the early phase of growth. The *eut* genes were found to be significantly up-regulated in AIEC strain LF82 when bile salts were added to the culture medium (Fig. 4B), and significantly up-regulated in comparison to the MG1655 strain (Fig. 4C). These data suggest that AIEC strain LF82 degrades EA more efficiently than other *E*. *coli* in the presence of bile salts. The utilization of EA could provide a nutritional advantage for AIEC. To test this hypothesis, competitive assays were performed between LF82 strain, its isogenic *eutB* mutant and the strain MG1655. The LF82 strain was able to grow in the presence of EA much more effectively than its isogenic mutant or the MG1655 strain (Fig. 4D). Interestingly, competitive index values close to 1 were obtained when the LF82Δ*eutB* and MG1655 strains were co-incubated, indicating that EA present in mM9b-EA was poorly used by the *E*. *coli* strain MG1655. *Enterococci* are prominent members of the normal human intestinal microflora. To analyze the capacity of *Enterococcus* to catabolize EA, 4 strains (*Enterococcus faecium* and three strains of *Enterococcus faecalis*) were incubated in mM9-EA with bile salts, and no growth was observed while they grow in mM9 supplemented with glucose and NH_4_Cl (data not shown). Altogether, these data suggest that, in the presence of bile salts, EA might be a privileged substrate for AIEC.

**Figure 5:**
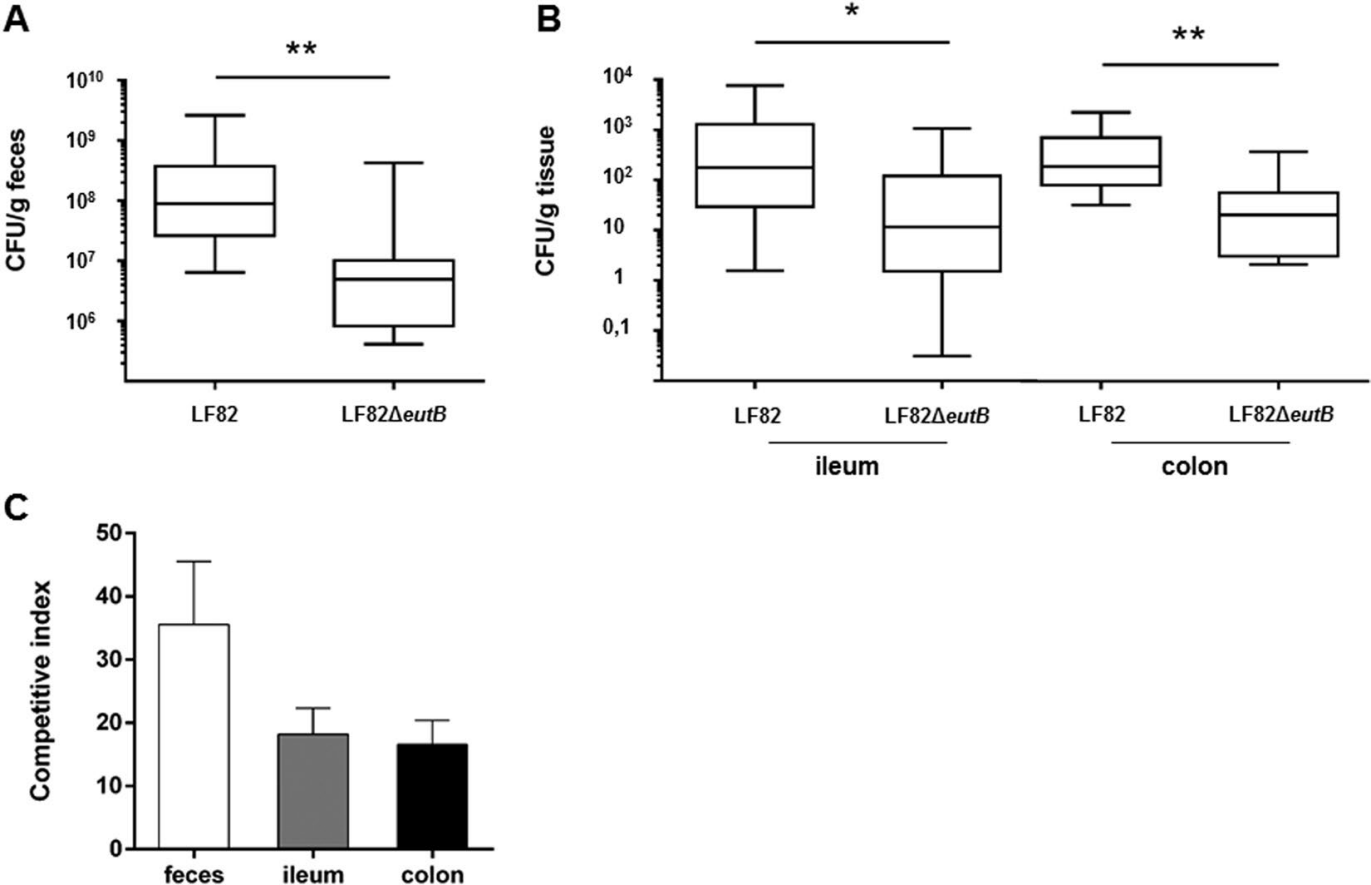
*eutB* favors AIEC strain LF82 gut colonization in mice. Mice (n = 10) were orally co-infected with 0.5 × 10^9^ of the LF82 strain and 0.5 × 10^9^ of the LF82Δ*eutB* mutant. (**A**) Feces were collected three days post-infection, and LF82 and LF82Δ*eutB* strains were counted. (**B**) Ileal and colonic mucosal-associated LF82 and LF82Δ*eutB* bacteria were quantified three days post-infection. Boxes are extended from the 25^th^ to 75^th^ percentiles. All the values and the median are represented. (**C**) Competitive index between LF82 and LF82Δ*eutB* bacteria in feces, ileum and colon collected three days post-infection. Values are means ± SEM. Statistical analysis was performed with the Mann-Whitney test; *p < 0.05; **p < 0.01.

We next evaluated the capacity of AIEC LF82 to use EA to colonize the gut. Mice, with colitis caused by dextran sulfate sodium (DSS), were orally challenged with an equal mixture of the LF82 strain and the LF82Δ*eutB* isogenic mutant. Quantification of bacteria in stool samples and ileal and colonic tissues revealed approximatively a one-log reduction in the LF82Δ*eutB*-AIEC mutant compared with wild-type LF82 (Fig. 5A,B). Analysis of the competitive index of LF82 bacteria growth compared with isogenic mutant growth revealed a median competitive index of 35, 18 and 16 for stool, ileum and colon, respectively (Fig. 5C).

These results indicate that AIEC may gain a competitive advantage in the intestine by using EA.

### An increased intracellular acetyl-CoA pool was induced by bile salts

Acetyl-CoA can be produced by EA degradation. EA is converted to ammonia and acetaldehyde, which is converted to acetyl-CoA by EutE, a component that is significantly increased in the presence of bile salts (RNA-seq data: log_2_ = 10.4; Table 1). Inside bacteria, acetyl-CoA is central to energy generation and to several biosynthetic pathways that utilize the activated two-carbon acetyl unit. However, EA is not the exclusive source of acetyl-CoA generation. Through its oxidative decarboxylation, pyruvate is a major source of acetyl-CoA. Pyruvate is usually formed by glycolysis to be oxidized by the the tricarboxylic acid (TCA) cycle. Genes involved in glycolysis and pyruvate decarboxylation (*aceE*, *aceF and lpd*) were not up-regulated during exposure to bile salts (Table 2). Phophoenolpyruvate, which is the precursor of pyruvate in glycolysis, was present in the same amounts in bacterial cultures with or without bile salts (30.3 ± 2.1 and 29.2 ± 4.9 mg/L, respectively; p = 0.886), confirming that the glycolysis pathway was not up-regulated by bile salts. Acetate, for example, could also lead to the production of acetyl-CoA. Interestingly, genes encoding citrate lyase (*citD*, *citE* and *citF*) were up-regulated under bile conditions (Table 3). The citrate lyase converts citrate to oxaloacetate and acetate (Fig. S3). One of two distinct pathways by which *E*. *coli* activates acetate to acetyl-CoA is the activation of acetyl-CoA synthetase (Acs)^15^. The *acs* gene was significantly up-regulated in the presence of bile salts (fold change: 2.6). Up-regulation of *eutE* and *acs* genes suggested that AIEC strain LF82 is able to produce a pool of acetyl-CoA under bile stress.

**Table 2:** Variations of the expression of genes involved in glycolysis, TCA cycle and formation of acetyl-coA.

Name	Product	Gene size	RNA-seq values (minimal medium)	RNA-seq values (bile salts)	Fold change	Fold change	p value
		(bp)	RPKM	RPKM		(Log2)	
*aceE*	Pyruvate dehydrogenase E1	2663	6420	11797	−1.8	−0.88	1.188E-02
*aceF*	Dihydrolipoyllysine-residue acetyltransferase	1892	2620	3886	−1.5	−0.57	6.618E-02
*ackA*	acetate kinase	1202	2740	4056	−1.5	−0.57	7.020E-02
*acs*	acetyl-coenzyme A synthetase	1958	2188	837	**2.6**	**1.39**	1.897E-04
*eno*	Enolase	1298	9777	15019	−1.5	−0.62	6.921E-02
*fbaA*	Fructose-bisphosphate aldolase class 2	1079	3314	3828	−1.2	−0.21	4.847E-01
*fbaB*	Fructose-bisphosphate aldolase class 1	1124	943	837	1.1	0.17	4.857E-01
*fbp*	Fructose-1.6-bisphosphatase	998	3314	5750	−1.7	−0.79	1.384E-02
*fumA*	Fumarate hydratase class I. aerobic	1646	7038	9348	−1.3	−0.41	1.869E-01
*fumB*	Fumarate hydratase class I. anaerobic	1646	771	415	1.9	0.90	5.607E-05
*fumC*	Fumarate hydratase class II	1403	1122	884	1.3	0.34	1.468E-01
*gapA*	glyceraldehyde-3-phosphate dehydrogenase A	995	25736	36899	−1.4	−0.52	1.405E-01
*glk*	glucokinase	965	832	912	−1.1	−0.13	6.037E-01
*gltA*	Citrate synthase	1283	7479	15175	**−2.0**	**−1.02**	7.423E-04
*gpmA*	bisphosphoglycerate-dependent phosphoglycerate	752	10216	19188	−1.9	−0.91	4.772E-03
*gpmM*	bisphosphoglycerate-independent phosphoglycerate	1544	1282	1810	−1.4	−0.50	7.967E-02
*icd*	Isocitrate dehydrogenase [NadP]	1250	17987	30473	−1.7	−0.76	1.944E-02
*lpd*	Dihydrolipoyl dehydrogenase	1424	10039	20656	−2.1	−1.04	1.135E-03
*mdh*	Malate dehydrogenase	938	5402	8506	−1.6	−0.66	2.854E-02
*pfkA*	6-phosphofructokinase isozyme 1	962	1283	4244	−3.3	−1.72	1.369E-11
*pgi*	glucose-6-phosphate isomerase	1649	4480	5251	−1.2	−0.23	4.652E-01
*pgk*	Phosphoglycerate kinase	1163	2762	4408	−1.6	−0.67	3.299E-02
*pta*	Phosphate acetyltransferase	2144	1355	1764	−1.3	−0.38	1.523E-01
*pykA*	Pyruvate kinase II	1442	1163	1360	−1.2	−0.23	3.798E-01
*pykF*	Pyruvate kinase I	1412	2019	5004	**−2.5**	**−1.31**	7.772E-07
*sdhA*	Succinate dehydrogenase flavoprotein subunit	1766	8036	14377	−1.8	−0.84	1.123E-02
*sdhB*	Succinate dehydrogenase iron-sulfur subunit	716	4221	6984	−1.7	−0.73	2.521E-02
*sdhC*	Succinate dehydrogenase cytochrome b556 subunit	389	4494	8916	−2.0	−0.99	1.946E-03
*sdhD*	Succinate dehydrogenase hydrophobic membrane	347	2126	3986	**−1.9**	**−0.91**	6.527E-04
*sucA*	2-oxoglutarate dehydrogenase E1 component	2801	18453	38038	−2.1	−1.04	1.922E-03
*sucB*	Dihydrolipoyllysine-residue succinyltransferase	1217	9625	19122	−2.0	−0.99	5.780E-03
*sucC*	Succinyl-CoA synthetase beta chain	1168	7109	12688	−1.8	−0.84	1.840E-02
*sucD*	Succinyl-CoA ligase [adP-forming] subunit α	869	9683	12034	−1.2	−0.31	3.924E-01
*tpiA*	Triosephosphate isomerase	767	4649	4399	1.1	0.08	8.098E-01

^**1**^Values shown in bold are significant.

**Table 3:** Variations of the expression of genes involved in citrate degradation and methyl-citrate pathway.

Name	Product	Gene size	RNA-seq values (minimal medium)	RNA-seq values (bile salts)	Fold change	Fold change	p value
		(bp)	RPKM	RPKM		(Log_2_)	
*acnA*	aconitate hydratase 1	2675	1824	2317	−1.3	−0.34	2.142E-01
*acnB*	aconitate hydratase 2	2597	11663	19016	−1.6	−0.70	2.362E-02
*acs*	acetyl-coenzyme A synthetase	1958	2188	837	**2.6**	1.39	1.897E-04
*citC*	Citrate-lyase ligase	1058	326	588	**1.8**	0.85	8.689E-04
*citD*	Citrate lyase acyl carrier protein	296	5	50	**9.1**	3.18	3.731E-10
*citE*	Citrate lyase β subunit	908	48	332	**7.0**	2.80	1.628E-17
*citF*	Citrate lyase α chain	1532	55	362	**6.6**	2.72	8.791E-16
*citG*	dephosphocoenzyme-A	878	32	183	**5.6**	2.49	5.951E-14
*citT*	Citrate carrier	1463	271	732	**2.7**	1.43	2.076E-09
*citX*	Holo-citrate lyase synthase	551	5	59	**10.6**	3.41	1.042E-13
*ttdA*	L-tartrate dehydratase subunit α	908	237	49	**4.8**	2.27	2.511E-13
*ttdB*	L-tartrate dehydratase subunit β	605	317	66	**4.8**	2.27	6.822E-12
*ttdR*	transcriptional activator	932	635	341	**1.9**	0.90	5.398E-04
*ttdT*	tartrate carrier	1463	724	312	**2.3**	1.21	1.002E-06
*prpR*	operon regulatory protein	1586	192	435	**2.3**	1.19	1.071E-04
*prpB*	Methylisocitrate lyase	890	131	3094	**23.6**	4.56	1.828E-37
*prpC*	2-methylcitrate synthase	1169	106	1313	**12.3**	3.62	3.179E-30
*prpD*	2-methylcitrate dehydratase	1451	66	609	**9.3**	3.21	2.654E-26
*prpE*	Propionate-CoA ligase	1886	80	786	**9.8**	3.30	5.999E-33

^1^Values shown in bold are significant.

**Figure 6:**
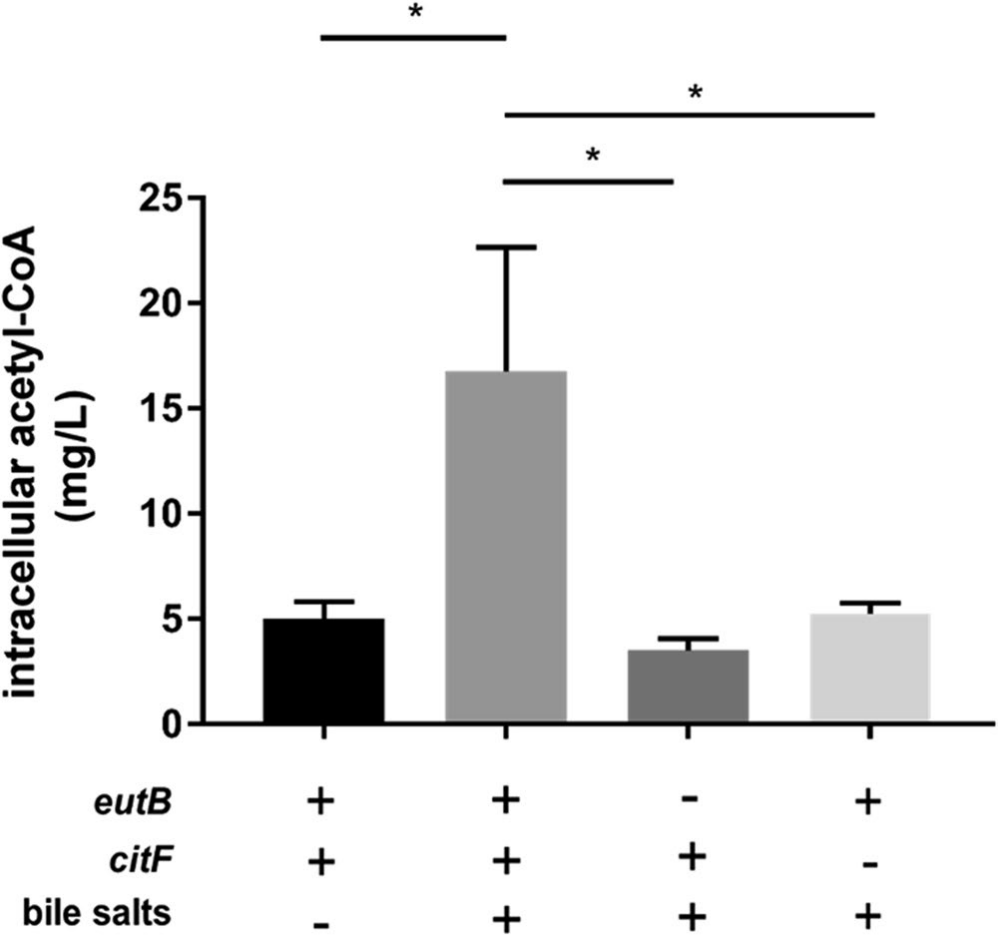
Bile salts increase the intracellular acetyl-CoA pool in the LF82 strain. LF82 and its mutants LFΔ*eutB* and LFΔ*citF* were grown in mM9 with 20% LB with or without bile salts. Values are means ± SEM of at least three independent experiments. Statistical analysis was performed with a Mann-Whitney test; *p < 0.05.

In order to test this hypothesis, the intracellular acetyl-CoA concentration was measured from bacterial cultures. The intracellular acetyl-CoA pool of LF82 strain increased when bile salts were added (5.2 ± 0.6 to 17.5 ± 4.3 mg/L; p = 0.0286) but not in mutants defective for ethanolamine ammonia lyase (LF82*ΔeutB*) or deficient for citrate lyase (LF82*ΔcitF*) (4.1 ± 0.3 and 5.9 ± 0.5 mg/L, respectively) (Fig. 6).

These observations confirmed that EA and citrate degradation pathways activated upon bile salts exposition lead to acetyl-CoA production by the AIEC strain LF82. In contrast, the intracellular acetyl-CoA pool of the strain MG1655 slightly decreased in the presence of bile salts (7.2 ± 0.8 to 4.3 ± 0.8 mg/L). The *eutB* and *citF* genes were found to be significantly down-regulated in the strain MG1655 grown in the presence of bile salts (12.5 and 4.2-fold, respectively; p = 0.0286). These data reinforce the fact that this strain and the AIEC strain LF82 have a different metabolism in the presence of bile salts.

### Induction of 1,2-propanediol degradation and methyl-citrate pathway by bile salts

The results of the RNA-seq analysis showed that the entire group of genes (*pdu* operon) was induced in the presence of bile salts (Table 4) suggesting the formation of propionyl-CoA from 1,2-propanediol (Fig. S3). Interrestingly, *pdu* operon is significantly correlated with the AIEC pathotype^13^. *E*. *coli* is known to ferment fucose and rhamnose to 1,2-propanediol^16^. Genes involved in the catabolism of rhamnose were up-regulated in the presence of bile salts (Table 4). Propionyl-CoA can also derive from propionate. Interestingly, the *prpE* gene encoding the enzyme that converts propionate to propionyl-CoA^15^ was significantly up-regulated by 9.8-fold (Table 3). The propionyl-CoA is the precursor of 2-methylcitrate, the coactivator needed for transcriptional activation of the *prpBCDE* operon by the PrpR activator protein. RNA-seq data analysis revealed that genes involved in the methyl-citrate pathway (*prpB*, *prpC*, *prpD*, *prpE*, *prpR*) were strongly up-regulated (2.3 to 23.6-fold), with the exception of *acnB*, a gene that is constitutively expressed (Table 3). The methyl-citrate cycle converts propionyl-CoA to pyruvate and succinate (Fig. S3). Thus, pyruvate might be produced from 1,2-propanediol *via* a pathway that is activated under bile conditions in AIEC strain LF82.

**Table 4:** Variations of the expression of genes involved in rhamnose and 1,2-propanediol degradation.

Name	Product	Gene size	RNA-seq values (minimal medium)	RNA-seq values (bile salts)	Fold change	Fold change	p value
		(bp)	RPKM	RPKM		(Log_2_)	
*rhaA*	L-rhamnose isomerase	1259	376	39	**9.7**	3.27	2.279E-24
*rhaB*	Rhamnulokinase	1469	452	65	**7.0**	2.80	3.564E-19
*rhaD*	Rhamnulose-1-phosphate aldolase	824	554	303	1.8	0.87	3.341E-03
*fucO*	propanediol oxydoreductase	1151	510	234	**2.2**	1.12	5.724E-06
*pduB*	polyhedral bodies	812	71	203	**2.8**	1.51	3.202E-07
*pduC*	glycerol dehydratase large subunit	1664	251	532	**2.1**	1.08	3.910E-06
*pduD*	diol dehydratase medium subunit	668	110	236	**2.2**	1.11	5.478E-04
*pduE*	diol dehydratase small subunit	518	39	118	**3.1**	1.62	7.898E-06
*pduG*	diol dehydratase reactivation	1832	185	494	**2.7**	1.42	8.661E-08
*pduH*	diol dehydratase reactivation	350	71	174	**2.4**	1.29	4.274E-05
*pduJ*	polyhedral bodies	275	42	135	**3.2**	1.69	2.362E-07
*pduK*	polyhedral bodies	422	16	59	**3.7**	1.89	1.644E-06
*pduL*	Phosphate propanoyltransferase	632	53	134	**2.6**	1.35	5.949E-05
*pduM*	propanediol utilization protein	491	78	233	**3.0**	1.59	2.260E-07
*pduN*	polyhedral bodies	275	48	90	1.9	0.91	1.456E-02
*pduO*	propanediol utilization: B12 related	1007	106	261	**2.5**	1.30	6.990E-06
*pduP*	propionaldehyde dehydrogenase	1112	81	211	**2.6**	1.39	2.872E-06
*pduQ*	Propanol dehydrogenase	1343	125	416	**3.3**	1.74	3.516E-09
*pduT*	polyhedral bodies	554	55	157	**2.8**	1.51	1.503E-05
*pduU*	polyhedral bodies	350	95	102	1.1	0.11	7.703E-01
*pduV*	propanediol utilization protein	443	188	152	−1.2	−0.31	2.703E-01

^1^Values shown in bold are significant.

### Interactions between metabolic pathways

To obtain 2-methylcitrate and subsequently pyruvate, propionyl-CoA must be condensed with oxaloacetate by the PrpC enzyme (Fig. S3). Interestingly, *prpC* mRNA exhibited a 12.3-fold increase in LF82 upon bile salt exposure. Oxaloacetate may have several origins, including the oxidation of L-malate. However, the gene encoding malate dehydrogenase was not deregulated in the presence of bile salts. Oxaloacetate is also derived from tartrate and citrate fermentation. RNA-seq analysis revealed that genes involved in those fermentations were induced in the presence of bile salts (Table 3). Interestingly, the intracellular oxaloacetate concentration of the LF82 strain significantly increased when bile salts were added to the culture medium (1.4 to 41.9 mg/L; *p* = 0.029), showing that oxaloacetate was available in the cell. Thus, bile salts may favor concomitant activation of (i) tartrate and citrate pathways, leading to the formation of oxaloacetate, (ii) rhamnose, propionate and 1,2-propanediol degradation, leading to the formation of propionyl-coA and subsequently the (iii) methylcitrate pathway leading to pyruvate (Fig. S6). Pyruvate can be transformed to acetyl-CoA by formate acetyltransferase II. Since *pflD* encoding formate acetyltransferase II was up-regulated 4.5-fold, pyruvate may also contribute to the increased intracellular acetyl-CoA pool under bile conditions. Thus, our results suggest that, in the presence of bile salts, these pathways act together to favor the formation of acetyl-CoA and to provide an energetic advantage to AIEC LF82 strain (Fig. S6).

### Alternative metabolic pathways of LF82 strain in mice in the presence of bile salts

**Figure 7:**
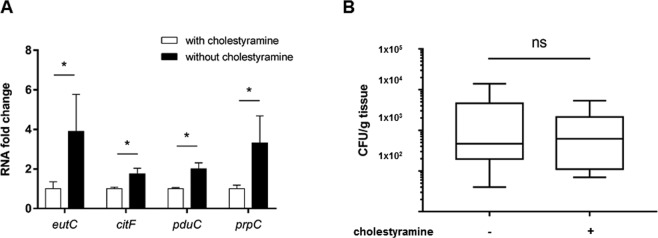
Alternative metabolic pathways in AIEC strain LF82 are induced in the small intestine of mice. Sixteen mice were inoculated with AIEC strain LF82. Cholestyramine (2%), a bile acid sequestrant, was added or not to the drinking water. (**A**) The mRNA of AIEC strain LF82 was extracted from the ileum of mice, and qPCR was performed for representative genes of major metabolic pathways that we had identified in this study (ethanolamine and 1,2-propanediol utilization, citrate degradation and methyl-citrate pathway). (**B**) Ileal mucosal-associated LF82 bacteria from mice treated or not treated with cholestyramine (2%) were quantified. Values are means ± SEM. Statistical analysis was performed with a Mann-Whitney test; *p < 0.05.

Our RNA-seq data clearly indicate that bile salts activate *in vitro* secondary metabolic pathways in AIEC strain LF82. As a test to elucidate whether bile salts are an *in vivo* metabolic activator of EA and 1,2 propanediol utilization and citrate and methyl-citrate pathways, the bile acid sequestrant cholestyramine was administred to C57BL/6 J mice. Cholestyramine decreases bile-acid pool size and is associated with decreased *Ibabp* and *Shp* levels in the ileum^17,18^. Down-regulation of these host genes in the group treated with cholestyramine indicates that the treatment was efficient (additional file 1: Fig. S7). The levels of bacterial mRNA extracted from the ileum of untreated mice were significantly increased in comparison with those treated with cholestyramine (Fig. 7). This observation strengthens the hypothesis that bile salts in the small intestine alter the metabolism of AIEC strain LF82 by inducing alternative metabolic pathways.

## Discussion

In this study, global RNA sequencing (RNA-seq) of AIEC reference strain LF82 has been used to decipher the impact of bile salts on the expression of metabolic bacterial genes. The results revealed a global up-regulation of genes involved in degradation and a down-regulation of those implicated in several biosynthetic pathways. The following four pathways were most noteworthy: ethanolamine utilization, citrate and 1–2 propanediol degradation, and methylcitrate pathway. Importantly, in LF82 bacteria isolated from the ileal mucosa of infected mice, the mRNA level of genes involved in these pathways were increased in comparison to mice treated with cholestyramine resin, which is a bile acid sequestrant, suggesting that these pathways are activated *in vivo* under bile conditions. Our results indicate that activation of these secondary metabolic pathways in LF82 AIEC favor the formation of acetyl-CoA (Fig. S6). Acetyl-CoA is a potential energy source because it can be converted to acetyl-PO4^2−^. The EutD phosphotransacetylase, for which the gene was up-regulated upon exposure to bile salts, can modify acetyl-CoA into acetyl-PO4^2−^. This enzyme is a more efficient phosphotransacetylase than *E*. *coli* Pta, which has activity linked to acetate excretion and to the operation of the Tricarboxylic Acid cycle^19^. Thus, the present study provides insight into the pathways favored by AIEC LF82 for optimal energy generation.

Comparative genomic analysis of AIEC strains from different collections (Europe, North America and Australia) show that this pathobiont does not harbour an exclusive molecular signature^20,21^, suggesting that AIEC strains have evolved from commensal *E*. *coli* by different mechanisms to favour their implantation in genetically susceptible CD patients. Our results identified two pathways, ethanolamine utilization and 1–2 propanediol degradation, that may be associated with the metabolic adaptation of the AIEC strains to their environments. The propanediol utilization (*pdu*) operon, which is prevalent among enteropathogenic bacteria such as *Salmonella*, *Yersinia*, *Listeria*, and *Clostridium perfringens*, is significantly correlated with the AIEC pathobiont^22–24^. In AIEC, the presence of *pduC*, which is significantly up-regulated in the presence of bile salts in our study, has been correlated with increased cellular invasion and bacterial persistence^13^. The utilization of 1,2-propanediol is therefore associated with virulent strains, and its utilization in this redirected metabolism in AIEC strain LF82 strengthens the hypothesis that this compound is important for the virulence of bacteria. Interrestingly, we demonstrated that ethanolamine can be used as a source of nitrogen and acetyl-CoA by the LF82 AIEC and confers a growth advantage to AIEC strains. It has been shown in the same way that the pathogenic *E*. *coli* O157:H7 strain EDL933 uses EA as a nitrogen source in the bovine intestine, whereas EA is poorly metabolized by the endogenous microbiota of the bovine small intestine, including commensal *E*. *coli*^25^. EA is abundant in the intestinal tract due to the turnover and exfoliation of enterocytes as well as the high concentration of bacterial cells^26^. However, EA does not contribute to the colonization of all gut bacteria. EA catabolism in *Enterococcus facecalis* modestly reduces gut colonization efficiency^27^. The ability of AIEC to utilize EA might therefore provide them with a competitive advantage to successfully colonize the intestinal mucosa as observed in the present study with LF82 in a mouse colitis model. Recently, it has been shown that uropathogenic *Escherichia coli* (UPEC) ethanolamine metabolism is required for effective bladder colonization in mice and that UPEC upregulates the expression of ethanolamine utilization genes during uncomplicated UTIs in humans^28^. Thus, we can speculate that optimization of the use of EA might be a metabolic adaptation of pathogenic *E*. *coli*.

EA is not only important for nitrogen metabolism but that it is also used as a signaling molecule in cell-to-cell signaling to activate virulence gene expression in EHEC^29,30^. The *eut* operon encodes the transcriptional regulator EutR that was found to bind promotors and directly activate the expression of the locus of enterocyte effacement (LEE) in EHEC^31^. Interrestingly, EA promotes also expression of fimbriae in EHEC, in particular of long polar fimbriae (LPF)^30^. AIEC strains express LPF, which are known to target Peyer’s patches in a mouse CD model^32^. AIEC might therefore exploit EA as a metabolite but also coopt EA as a signaling molecule to recognize gastrointestinal environment and promote virulence expression. Further work is required to establish this. Nevertheless, the above-described accessory metabolic pathways may interact with synthetic pathways involved in biofilm formation. Indeed, these metabolic pathways can lead to the formation of acetate and pyruvate, which may participate in the formation of side chains of colanic acid. RNA-seq data analysis showed a global increase in the transcription of genes involved in the biofilm formation under bile conditions (genes involved in the formation of the colanic acid and of curli fimbriae, gene clusters encoding group 2 capsule^33^, the *auf* and *yeh* genes involved in adhesion^34^, …) (additional file 1: Table S2). In consistent with these data, the crystal violet assay revealed an increased of biofilm biomass and an autoaggregation of LF82 bacteria in presence of bile salts (additional file 1: Fig. S8). Thus, bile salts might favor AIEC gut colonization by inducing biofilm formation. Most strains of *E*. *coli*, *Salmonella* and *Shigella* forms a biofilm in the presence of bile salts; it is an important resistance mechanism for many bacterial pathogens^35,36^. Moreover, virulence factor expression of enteric pathogens and AIEC, is regulated during exposure to bile salts^9,10,12^. Given the dual control of resistance to bile salts and virulence factor expression in the presence of bile salts, it has been proposed that the enteric bile salt-induced biofilm is a transient phenotype that would allow a more rapid dispersion as the bacteria reach the epithelial cell surfaces in the small intestine^36^. The metabolic adaptation of AIEC presented in this work might be associated with this transient phenotype.

Studies examining the interactions between bacteria and bile have frequently been performed with cholic acid and deoxycholic acid, which are among the most abundant in the intestine^37^. Since more than 20 bile acid metabolites can be produced from primary bile acids by the gut microbiota, the use of only two bile acids in our this study has limitation and needs further investigation. The study of Chassaing et *al*.^9^ reported that the concentration of bile salts has no significant effect on *lfp* transcription, in contrast to the chemical nature of bile salts. That might be similar in our study. CD-associated intestinal dysbiosis leads to modifications in the luminal bile salt composition^38^. Thus, it is possible that the expression of AIEC genes (*eut*, *pdu*, *lpf…*) in the gut of CD patients is different from that of healthy subjects. In contrast to the study by Chassaing *et al*., we did not find a significant difference in LF82 *lpf* expression when LF82 was grown for 24 h at 37 °C in a minimal medium (M9) with or without bile salts, suggesting that bacterial culture medium is also crucial for *lpf* gene expression. Further studies, which take these variables into account, are therefore necessary.

To conclude, in conjunction with previous work, our results support the notion that AIEC under bile conditions modulate expression of their virulence genes as well as secondary metabolic pathway genes to overcome nutritional limitations and compete with the indigenous microbiota. Thus, our study highlight a metabolic adaptation of AIEC to bile salt exposure that may favor their colonization.

## Methods

### Bacterial strain and bacterial growth conditions

The bacterial strains used in this study are listed in additional file 1 (Methods, Table S3). The ability of *E*. *coli* LF82 to grow with bile salts and ethanolamine (EA) was tested on minimal medium mM9 as previously described^25^ (additional file 1: Methods). This medium was supplemented with bile salts (1%: 50% cholic acid sodium salt, 50% deoxycholic acid sodium salt, Sigma) when needed (mM9b).

For absolute quantification by RNA-seq, LF82 bacteria was grown for 24 h at 37 °C in mM9 medium supplemented with D-glucose (0.1% w/v) and NH_4_Cl (20 mM) in a 20 mM Tris buffer with a pH = 7.5, in the presence or absence of 1% bile salts. Each assay was performed in triplicate. Utilization of EA as a nitrogen source or as the sole carbon source was investigated in the the mM9b medium supplemented with EA hydrochloride (mM9b-EA, additional file 1: Methods). For the other growth curves and for qRT- PCR of the *eut* genes, the growth medium used was the mM9-EA supplemented with 0.01% glucose. Bile salts (1%) were added or not in this medium. For quantification of metabolites in bacteria, *E*. *coli* strains were grown at 37 °C to an OD600 of 0.7 in mM9 with 20% Lysogeny broth (LB). Then, bile salts (1%) were added or not for 4 h.

### RNA extraction, reverse transcription and quantitative PCR

The AIEC reference strain LF82 was grown for 24 h at 37 °C in a minimal medium with or without bile salts. Total RNAs were isolated from LF82 bacteria, and following rRNA depletion, the remaining RNA was reverse-transcribed into cDNA, fragmented and sequenced (for detailed Methods, see additional file 1: Methods). For qRT-PCR, extracted RNAs were quantified using a NanoPhotometer P-Class (Implen) and then reverse transcribed with random hexamers (Invitrogen) and NxGen M-MuLV Reverse Transcriptase (Lucigen) according to the manufacturer’s recommendations. RNAs were then amplified using specific primers to the *prpB*, *folX*, *pfkA*, *cfa*, *LF_715*, *eut*, *tufA* or *16S*r*RNA* genes (Aditional file 1: Methods, Table S1 and S4). Amplification of a single expected PCR product was confirmed by electrophoresis on a 2% agarose gel. qRT-PCR were performed using an Eppendorf Realplex with the following program: 95 °C for 5 min and 40 cycles of 95 °C for 15 s, 60 °C for 20 s and 72 °C for 40 s. For absolute qRT-PCR, the transcript levels in each sample were determined by absolute quantification using serial dilutions of PCR products as previously described^39^. Each experiment was performed in triplicate. For relative qRT-PCR, each gene expression was normalized to *tufA* using the 2^−ΔΔCt^ method.

### RNA-seq data analysis

The RNA-seq data analysis was performed using the R software, Bioconductor packages including edgeR and the SARTools package developed at PF2-Institut Pasteur^40^ (additional file 1: Methods).

### Construction and transcomplementation of mutants

Isogenic mutants of *E*. *coli* LF82 was generated by using the lambda red recombination system described by Datsenko and Chaveroche^41,42^ (additional file 1: Methods).

### Competition experiments

Precultures of *E*. *coli* strains (inoculated from a single colony) were incubated in LB with the appropriate antibiotic (32 mg/L amoxicillin for LF82 bacteria and 50 mg/L kanamycin for LFΔ*eutB*). The bacterial cultures were diluted 50-fold in LB broth and grown overnight at 37 °C without shaking. The mM9b-EA medium previously used was supplemented with glucose (0.1%), inoculated with approximately 5.10^7^ bacteria per milliliter of each of the two strains tested in competition assays and incubated at 37 °C without shaking. At each time point, the co-culture was diluted in phosphate-buffered saline (PBS) and spotted onto trypticase soy (TS) agar plates with and without antibiotic. Co-cultures of LF82/LFΔ*eutB* bacteria were spotted on plates without antibiotic and on plates containing kanamycin (50 mg/L). Co-cultures of LF82 or LFΔ*eutB*/MG1655 bacteria were spotted on plates without antibiotic and on plates containing amoxicillin (32 mg/L). The plates were then incubated overnight at 37 °C before counting the colony-forming units (CFU). The CFU counts of the antibiotic-sensitive strain for each competition assay were calculated by subtracting the number of CFU that were resistant to amoxicillin or kanamycin from the number of CFU counted on an agar plate without antibiotic. Each experiment was repeated three times, and a competitive index (CI) was calculated.

### Metabolite extraction

Bacteria were harvested by centrifugation (10,000 g for ten minutes at 4 °C). Quantified concentrations of metabolites were normalized to 3.10^10^ bacteria. The supernatant was stored at −80 °C, and the pellet was frozen at −20 °C, thawed on ice, resuspended in cold extraction buffer (10 mM phosphate buffer, 10 mM MgCl_2_, 1 mM EDTA) and then disrupted by ultrasonic treatment (five times for 30 seconds). The extract was clarified by centrifugation at 10,000 g for 10 min. After addition of DNase I, the supernatant extract was centrifuged at 4 °C, 10,000 g for 20 min, passed through a 0.2-µm filter and frozen to −80 °C.

### Metabolite quantification

LC/MS analyses were performed on a ThermoScientific UHPLC Ultimate 3000 RSLC coupled to an Orbitrap Q-Exactive analyser. The samples were diluted before the LC–MS analyses and injected directly in the LC–MS system without any further treatment. The calibration solutions were prepared from analytical standards dissolved in water. The UHPLC was equipped with a Luna Omega Polar C18 (100 × 2.1 mm; 1.6 µm) (Phenomenex) at 30 °C with a gradient of acetonitrile + 0.1% formic acid and water + 0.1% formic acid. The analyses were carried out in negative mode (ESI−) with a spray voltage at 3.0 kV.

### Murine model of gut colonization

The study was carried out in strict accordance with the recommendations of the Guide for the Care and Use of Laboratory Animals of the Université Clermont Auvergne. The animal protocol was approved by the committee for ethical issues, CEMEA Auvergne (Permit Number: CEMEAA, 2015032716314007), and all animals were used in accordance with the European Community Directive in the care and use of animals (86/609/CEE).

For *in vivo* competition assays, ten twelve-week-old C57BL/6 mice (body weight ≈26–28 g)^43^ were pretreated by administering oral antibiotics and 3% dextran sulfate sodium salt (Sigma) before being orally challenged with 10^9^ bacteria (50% LF82–50% LFΔ*eutB*) (Supplementary information in addition file 1: Methods). Three days after bacterial infection, fresh fecal pellets (100–200 mg) were collected from individual mice and resuspended in PBS (addition file 1: Methods). Then, the mice were anesthetized with isoflurane and then euthanized by cervical dislocation. Colonization of the two strains was studied by enumerating the mucosa-associated AIEC bacteria by homogenizing 0.5 cm of ileum and 1 cm of colon, beginning at 0.5 cm from the cecal junction, in sterile PBS solution.

To determine the bacterial mRNA levels in the ileum of mice, the mice were pretreated by administering oral antibiotics for four days. From the day of infection until sacrifice, 2% cholestyramine was mixed with the drinking water, as recommended for human patients, in the group of treated mice. This suspension was regularly resuspended. All animals were orally challenged with 10^9^ LF82 bacteria. The day after infection, the mice were anesthetized with isoflurane, euthanized by cervical dislocation and the ileum was removed.

### Statistical analysis

Statistical comparisons were performed using the Mann-Whitney nonparametric test. An analysis of variance (ANOVA) was performed for experiments with multiple treatment groups, followed by pairwise comparisons with Bonferroni’s multiple comparison tests. A *p* value less than 0.05 was considered statistically significant (*p < 0.05; **p < 0.01; and ***p < 0.001). For RNA-seq, mRNAs that were differentially expressed below the *p*-value threshold of 0.001 were considered significant.

## Supplementary information

Supplemental information

RNA-seq data
