# Supplementary material for: Metabolic adaptation of adherent-invasive Escherichia coli to exposure to bile salts
Source: Sci Rep. 2019 Feb 18;9:2175. doi: 10.1038/s41598-019-38628-1 (PMC6379400; doi:10.1038/s41598-019-38628-1)
Supplement: Supplementary file 1 — Supplemental information [file 41598_2019_38628_MOESM1_ESM.pdf]

Metabolic adaptation of adherent-invasive *Escherichia coli* to exposure to bile salts

Julien Delmas*[1,2]✉, Lucie Gibold*[1,2], Tiphanie Faïs[1,2], Sylvine Batista[1], Martin Leremboure[4], Clara Sinel[3], Emilie Vazeille[2], Vincent Cattoir[3], Anthony Buisson[2,5], Nicolas Barnich[2,6], Guillaume Dalmasso[2], Richard Bonnet[1,2]

Supplemental Methods

Bacterial strains

E. coli were isolated from ileal biopsies of patients. In this prospective multicentre study (8 centers), all the patients requiring ileocolonoscopy, regardless of the indication, were consecutively included between September 2015 and September 2016. The study was performed in accordance with the Declaration of Helsinki, Good Clinical Practice and applicable regulatory requirements. Study ethics approval was obtained on CPP Sud-Est 6, France (approval number AU 904). Suspicious *E. coli* colonies were identified by MALDI-TOF mass spectrometry (bioMérieux, France). Determination of *E. coli* strains as belonging to the AIEC pathovar was performed using the following criteria: (1) the ability to adhere to I-407 epithelial cells with an adhesion index equal or superior to 1 bacteria per cell, (2) the ability of the bacteria to invade I-407 with an invasion index equal or superior to 0.1% of the original inoculum [1].

Strain LF82, isolated from a patient with ileal CD, is the archetypal AIEC strain, belongs to the *E. coli* serotype O83:H1 [2] (additional file 1: Table S3). This strain contains cephalosporinase and can be selected on amoxicillin-containing agar plates. The non-pathogenic *E. coli* K-12 strain MG1655 does not display the AIEC phenotype.

Bacterial growth conditions

A modified M9 minimal medium (mM9) containing Na_2HPO_4 (48 mM), KH_2PO_4 (22 mM), and NaCl (8.5 mM) was supplemented with $MgSO_4$ (1 mM), $CaCl_2$ (0.1 mM), vitamin B12 (cyanocobalamin) (150 nM), vitamin B1 (thiamine, 5 mg/L) and trace metals (0.1 µM $ZnSO_4$, 0.045 µM $FeSO_4$, 0.2 µM $Na_2Se_2O_3$, 0.2 µM Na_2MoO_4, 2 µM $MnSO_4$, 0.1 µM $CuSO_4$, 3 µM $CoCl_2$ and 0.1 µM $NiSO_4$) (2 mL/L). This medium was supplemented with bile salts (1%: 50% cholic acid sodium salt, 50% deoxycholic acid sodium salt, Sigma) when needed (mM9b).

Utilization of EA as a nitrogen source was investigated by addition of EA hydrochloride (5 mM) and glucose (0.1 %) to the mM9b medium (mM9b-EA). To test the capacity of the LF82 strain to use EA as the sole carbon source, the mM9b medium was supplemented with EA hydrochloride (5 mM) and NH_4Cl (20 mM). For each condition, three lysogeny broth (LB) cultures were each started from a single colony and grown overnight at 37°C with aeration. Cells were pelleted by centrifugation, resuspended in medium with EA as the sole nitrogen or carbon source and diluted 50-fold in the corresponding medium. Cultures were then incubated at 37°C, and growth was monitored in three parallel cultures by following the optical density (λ=600 nm).

RNA extraction

Total RNAs were extracted from bacteria using a Direct-zol RNA MiniPrep kit (Zymo research) and treated with a Turbo DNA-free kit (Ambion) to remove any contaminating genomic DNA. DNase-treated RNA samples were purified with RNA clean and concentrator-25 (Zymo research). For RNA-seq analysis, purified RNAs were then quantified using a NanoDrop 1000 spectrophotometer (Thermo Scientific). The integrity of the results (RNA integrity number [RIN]) was assessed using an Agilent 2100 bioanalyzer. A Ribo-zero Magnetic kit for Gram-negative bacteria (Epicentre) was used according to the manufacturer’s recommendations to

remove the 23S and 16S rRNA from the total RNA samples. The samples were then purified using RNA clean and concentrator-5 (Zymoresearch). To evaluate the degree of rRNA depletion, the samples were analyzed using an Agilent 2100 bioanalyzer. The remaining RNA was sequenced using Illumina HiSeq 2500 technology with the genomic ProfileXpert platform (Claude Bernard University, Lyon, France). Three replicates from each experimental condition (with or without bile salts) were employed.

RNA-seq data analysis

We used RNA-seq to compare transcriptomes to understand the regulatory networks that control gene expression in AIEC strains during ileal colonization. Briefly, reads were mapped against the genomic sequence of *E. coli* LF82 (GenBank accession n° NC_011993; [3]). Reads mapped to several positions and reads mapped to rRNA were removed from further analysis. The number of reads overlapping each gene based on GenBank annotation was recorded. Reads from replicate samples were pooled, and the number of reads per gene was normalized according to the total number of reads in each library and the gene size. To normalize the expression of genes in different RNA-seq samples, values corresponding to the number of reads per kilobase per million mapped (RPKM) were calculated as follows: (number of reads for the gene x 10^9) / (total number of reads x size of the gene). A *p*-value adjustment for differentially expressed genes (DEGs) was performed to take into account multiple testing and control the false positive rate to a chosen level $\alpha < 0.001$. Products of DEGs were classified by functional category according to metabolism pathways of the Ecocyc database (http://ecocyc.org) [4]. The RKPM values for each gene were plotted and visualized as a circle using the Circos program [5]. Metabolic pathways were analyzed using Ecocyc [6].

A total of 53,900,605 reads from minimal medium and 56,327,631 reads from medium supplemented with bile salts were obtained for each cDNA library. Among them, 50,353,549

(93.4%) and 53,246,854 (94.5%) reads mapped to the genome of *Escherichia coli* LF82, which has a total size of 4,773,108 bp. The average numbers of reads per region were 462 (without bile salts) and 747 (with bile salts), with a coverage of 98.4% and 98.6% of the 4376 encoding DNA-encoding sequences (CDS) of *E. coli* LF82 represented by at least one single read. The absolute and relative distributions of reads in the two media for the annotated genes of the LF82 strain are shown in Fig. S1. A dendrogram used to represent all the samples shows the grouping of the replicates and a difference between the biological conditions. Box plots and the volcano plot are also represented to show the quality of the normalization and the differential expression of genes (Fig. S1). To assess the reliability of RNA-seq for determining the relative abundances of individual transcripts in the absence and presence of bile salts, we used absolute quantification of mRNAs for three up-regulated genes (*prpB*, *eutB* and *LF82_715*), two downregulated genes (*pfkA* and *cfa*) and one unchanged gene (*folX*) by qRT-PCR. The data for these genes provided an *r*2 value of 0.9708, confirming the data obtained by RNA-seq (Fig. S2; Table S1).

Construction and transcomplementation of isogenic mutants

Isogenic mutants of *E. coli* LF82 was generated by using the lambda red recombination system. *E. coli* LF82 was transformed with pKOBEG, a plasmid encoding the Red proteins that protect linear DNA from degradation in bacteria. The plasmid was maintained in bacteria at 30°C with 25 mg/l of chloramphenicol and 1 mM of L-arabinose. The Flp recognition target-flanked cassette harboring the kanamycin resistance cassette was generated by PCR from *E. coli* BW25141 with d-eutB-F/d-eutB-R, d-eutE-F/d-eutE-R and d-citF-F/citF-R primers (Table S2) and High Fidelity Platinum Taq polymerase (Invitrogen) according to the manufacturer's instructions. The PCR products were electroporated into previously glycerol-washed *E. coli* LF82. The resulting LF82Δ*eutB*, LF82Δ*eutE* and LF82 Δ*citF* isogenic mutants (Km^R) were

selected on LB agar containing 50 mg/L kanamycin. Replacement of the *eut* and *citF* genes by the kanamycin resistance cassette was confirmed by PCR. The kanamycin resistance cassette was then removed from LF82Δ*eutB* bacteria by the transient expression of the Flp recombinase from the pCP20 plasmid, creating the LF82Δ*eutB* (Km^S) strain.

The *eutB* gene was amplified by PCR from *E. coli* LF82 genomic DNA using eutBEcoR1-F and eutBBamH1-R primers (Table S2). The amplified DNA was purified with a NucleoSpin extract kit (Macherey-Nagel), digested with EcoRI and BamHI (New England Biolabs), and ligated to the EcoRI-BamHI-digested expression vector pBK-CMV (Agilent Technologies). This construct was electroporated into LF82Δ*eutB* (Km^S) electrocompetent strains and selected on Mueller Hinton agar containing 50 mg/L kanamycin. The presence of the *eutB* gene was confirmed by PCR. The construction was checked by double-stranded DNA sequencing (GATC biotech, Germany).

Murine model of gut colonization

For the *in vivo* experiments, we used C57BL/6 mice, which were housed in specific pathogen-free conditions in the animal care facility at the Université Clermont Auvergne, Clermont-Ferrand, France. For *in vivo* competition assays, ten twelve-week-old mice (body weight ≈26–28 g) were pretreated by administering oral amoxicillin (1 g/L), vancomycin (500 mg/L), metronidazole (500 mg/L) and neomycin (1 g/L) for four days, and 3% dextran sulfate sodium salt (Sigma) for the last day. At 24 h after stopping the antibiotic treatment, the animals were orally challenged with 10^9 bacteria (50% LF82 - 50% LFΔ*eutB*). Three days after bacterial infection, fresh fecal pellets (100–200 mg) were collected from individual mice and resuspended in PBS. After serial dilutions, the bacteria were enumerated by plating on TS agar medium containing amoxicillin to isolate the two bacteria and amoxicillin + kanamycin to isolate LFΔ*eutB.* The plates were then incubated overnight at 37°C before counting the CFU.

The CFU count of the LF82 strain for each mouse was calculated by subtracting the number of CFU that were resistant to kanamycin from the number of CFU counted on an agar plate containing only amoxicillin. Three days after infection, the mice were anesthetized with isoflurane and then euthanized by cervical dislocation. Colonization of the two strains was studied by enumerating the mucosa-associated AIEC bacteria by homogenizing 0.5 cm of ileum and 1 cm of colon, beginning at 0.5 cm from the cecal junction, in sterile PBS solution. Samples were plated on TS agar containing amoxicillin or amoxicillin + kanamycin and incubated overnight at 37°C.

Biofilm formation assay

Biofilm assays were used as previously described with some modifications [7,8]. Briefly, 6 µL of a 3-hour culture in mM9-EA medium supplemented or without 1% bile salts was inoculated into 144 µL of the same medium in a 96-well culture-treated polystyrene microtiter plate (Nunc). Wells filled with growth medium alone were included as negative controls. After 4 h and 30 min of incubation at 37°C, surface-adherent biofilm formation was measured by staining bound cells for 15 min with a 0.5% (w/v) aqueous solution of crystal violet. After rinsing with distilled water, the bound dye was released from the stained cells using 95% ethanol, and the OD at 540 nm was determined.

Autoaggregation assay

After an overnight culture at 37°C in M9 minimal medium supplemented with glucose (0.1%) and bile salts (1%) when needed, cells were diluted in the same medium and grown at 37°C to the exponential phase. Next, 20 µL of each culture was Gram-stained and visualized by microscopy.

Supplemental data

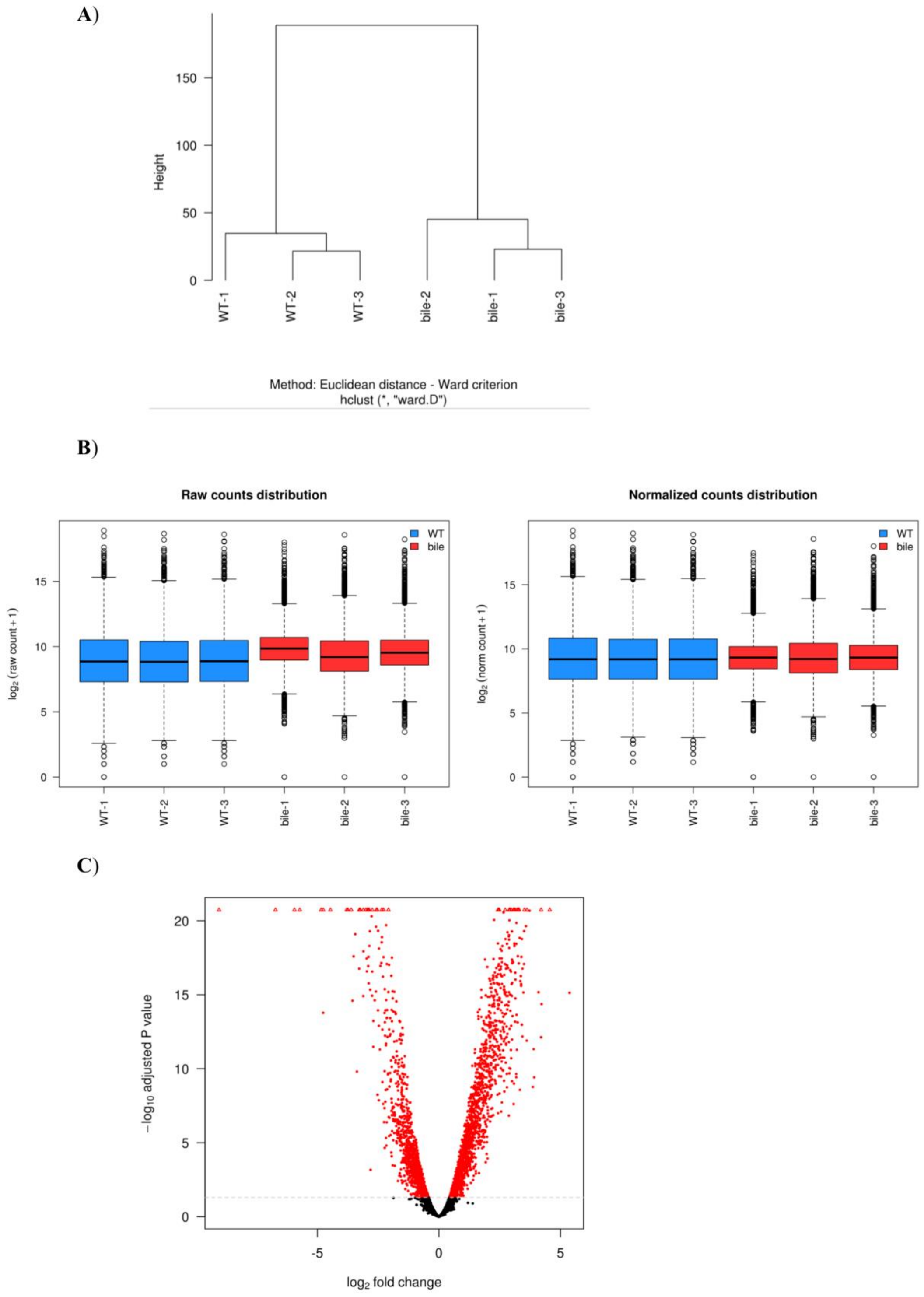

Figure S1: Global data retrieved by RNA-seq. (**A**) Dendrogram built based on the Ward criteria, which show variability in raw data within the experimental conditions. (**B**) Boxplots of raw (left) and normalized (right) data to assess the quality of the normalization process. (**C**) Volcano plot (bile salts containing medium *versus* medium) representing the log of the adjusted p value as a function of the log ratio of differential expression.

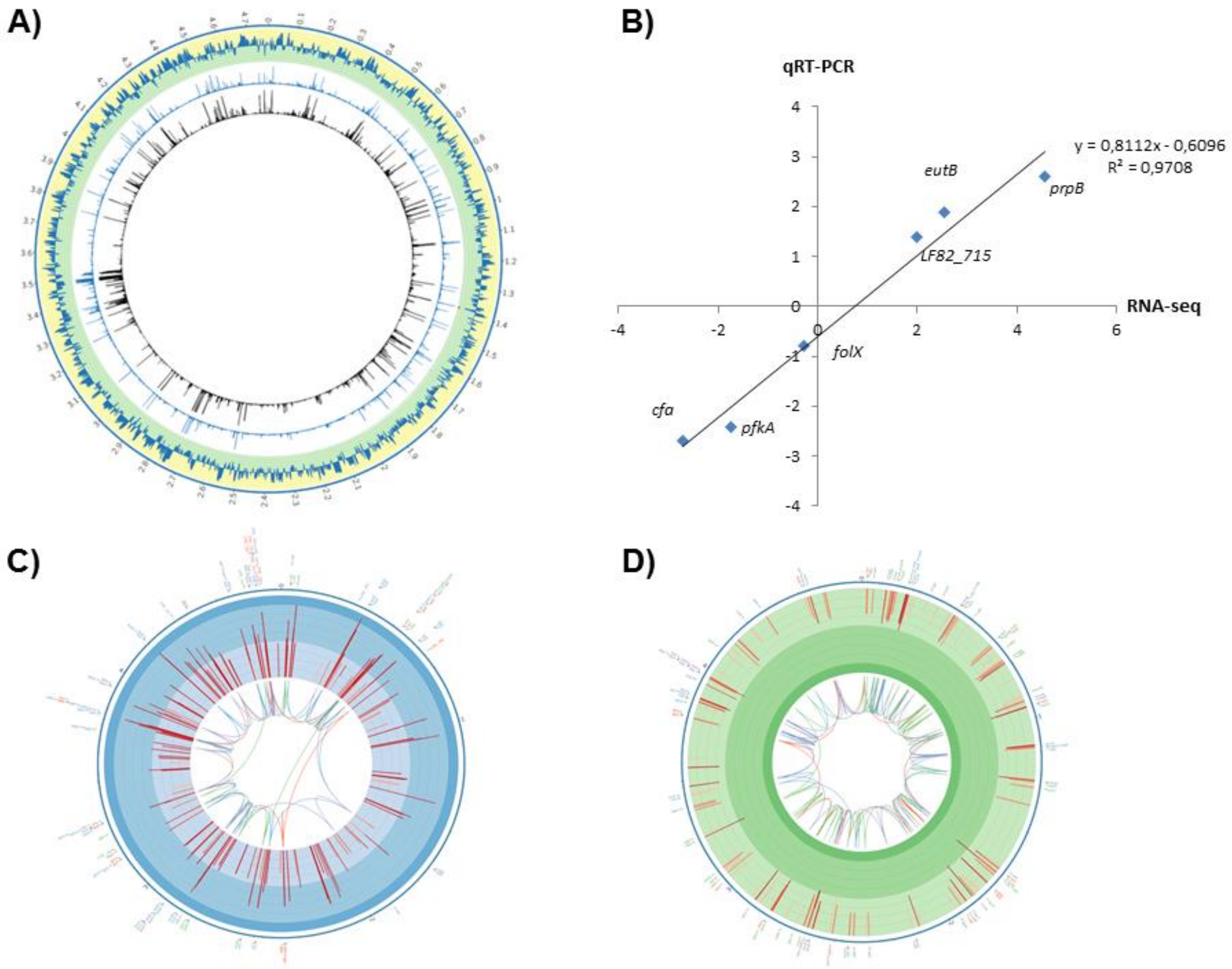

Figure S2: Analysis and validation of the RNA-seq experiments.

(**A**) Global analysis of transcript levels in *E. coli* LF82 by RNA-seq. The gray circular lines represent a $\log_2 = 1$ (i.e., RPKM bile salts containing medium/minimal medium ratio values) of each gene among the upregulated genes (yellow background) and downregulated genes (green background). Black and blue circles on a white background correspond to the expression of each gene (represented as RPKM values) in bacteria grown under minimal conditions and bile salts-containing medium, respectively. The outermost circle represents the 4,773,108 bp of the LF82 genome. (**B**) Validation of the RNA-seq results by qRT-PCR for selected genes. Mean $\log_2$ ratios of values determined in the qRT-PCR experiments are plotted against the mean $\log_2$ ratios of values determined in the RNA-seq experiments (Supporting Information Table S1). (**C**) Global analysis of increased transcript levels of genes involved in metabolism in *E. coli* LF82 with connections with each metabolic pathway. The gray circular lines represent a $\log_2 = 0.5$ (i.e., RPKM bile salts containing medium/minimal medium ratio values) of each gene among the upregulated genes. (**D**) Global analysis of decreased transcript levels of genes involved in metabolism in *E. coli* LF82 with associations with each metabolic pathway. The gray circular lines represent a $\log_2 = 0.5$ (i.e., RPKM bile salts containing medium/minimal medium ratio values) of each gene among the downregulated genes.

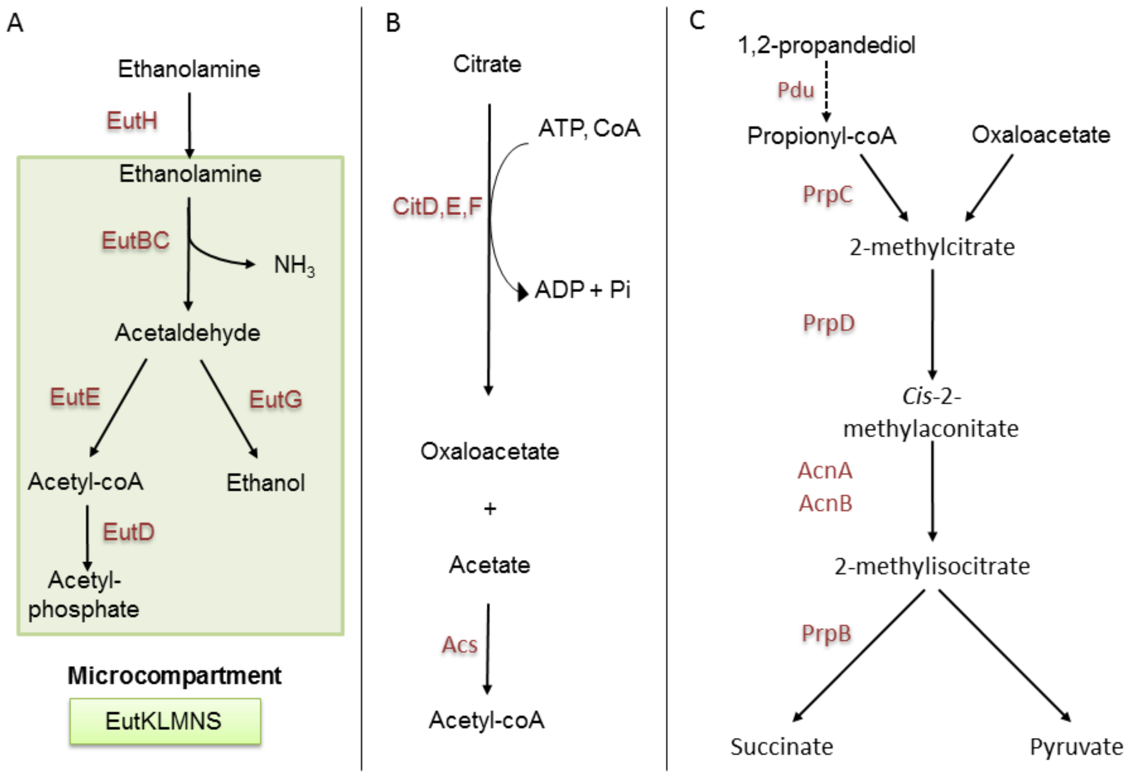

Figure S3: Schematic pathways of ethanolamine catabolism (A), citrate degradation (B) and the methylcitrate pathway (C).

(**A**) EutS, EutM, EutN, EutL and EutK are shell proteins constituting a carboxysome, an organelle that is required to conserve volatile metabolites and to concentrate the EA catabolic enzymes. Ethanolamine ammonia lyase, encoded by the genes *eutB* and *eutC*, converts EA to ammonia and acetaldehyde. This process requires the cofactor adenosylcobalamin, which is produced from cobalamin by the corrinoid cobalamin adenosyltransferase protein encoded by *eutT*. While ammonia can serve as a cellular source of reduced nitrogen, the acetaldehyde is further converted to acetyl-coenzyme A by an aldehyde oxidoreductase encoded by *eutE*, and it enters the carbon pool of the cell. In the case of acetyl-CoA accumulation, acetaldehyde can be converted to alcohol by another oxidoreductase encoded by *eutG*. (**B**) Citrate is cleaved to oxaloacetate and acetate by citrate lyase enzyme, which is a complex of three subunits. (**C**) The methylcitrate cycle is initiated by the synthesis of 2-methylcitrate from propionyl-CoA, and oxaloacetate. 2-methylcitrate is then converted into 2-methylisocitrate, which is subsequently split into pyruvate and succinate.

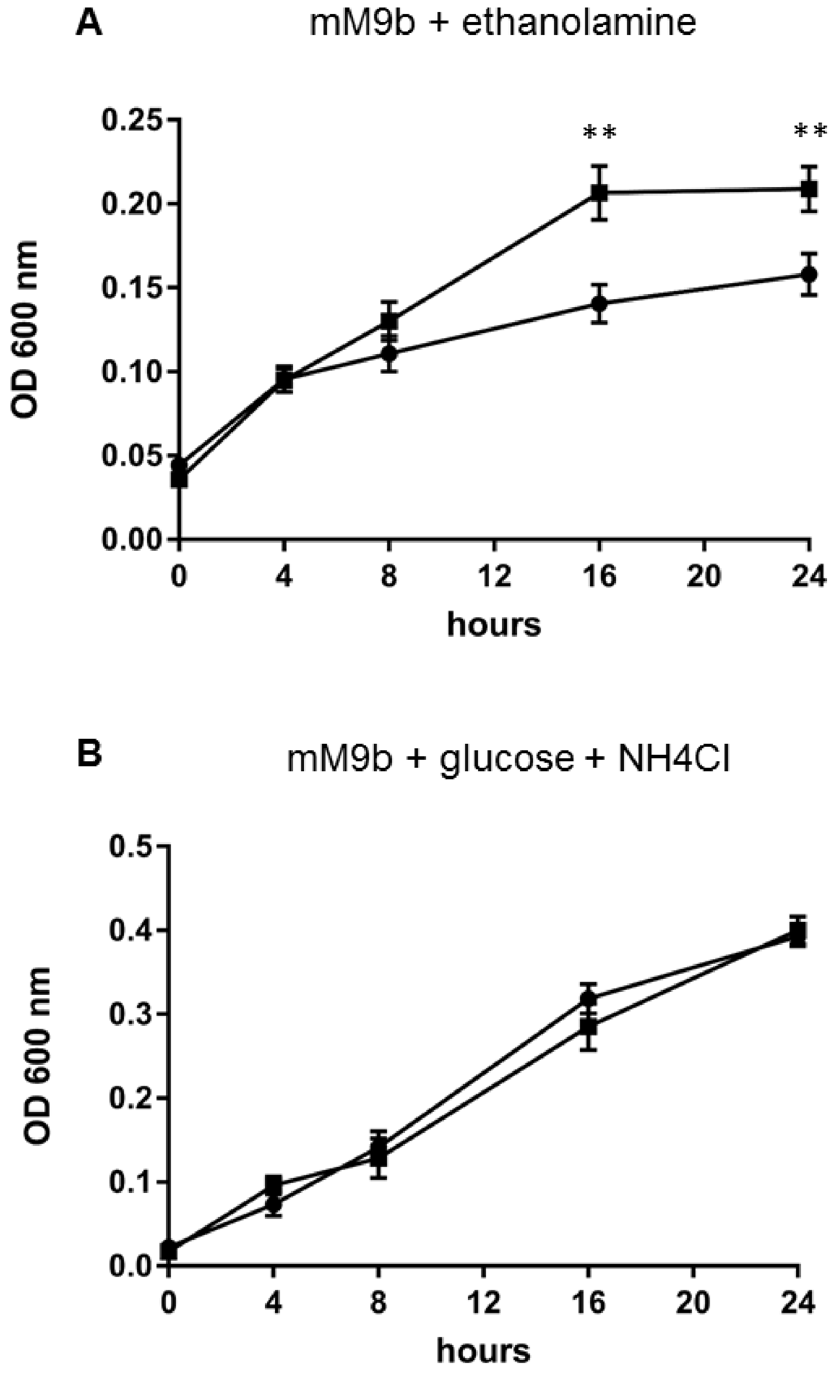

Figure S4: Comparison of growth of AIEC and non-AIEC strains in minimal medium.

Data are presented as mean values ± SEM of bacterial growth curves from AIEC (n=18) and non-AIEC strains (n=18) isolated of ileal mucosa of CD patients. (A) Bacterial growth curves of AIEC and non-AIEC strains in mM9b supplemented with ethanolamine. (B) Bacterial growth curves of AIEC and non-AIEC strains in mM9b supplemented with glucose and NH_4Cl. Statistical analysis was performed using a Student's t test; ** $p<0.01$.

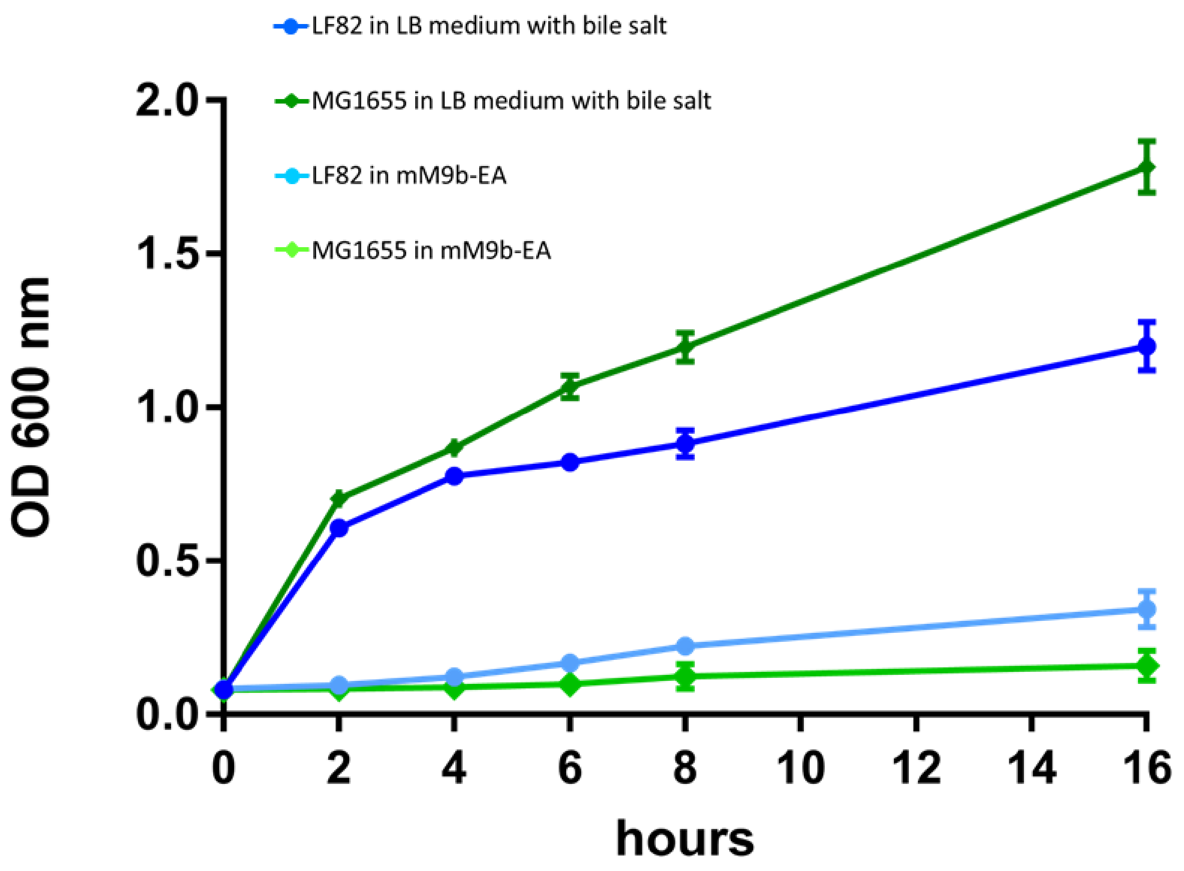

Figure S5: Bacterial growth curves of LF82 and MG1655 strains in LB and mM9-EA supplemented with bile salts. Values are means ± SEM of at least three independent experiments. The growth difference between both media was 68 and 90% for LF82 and MG1655, respectively.

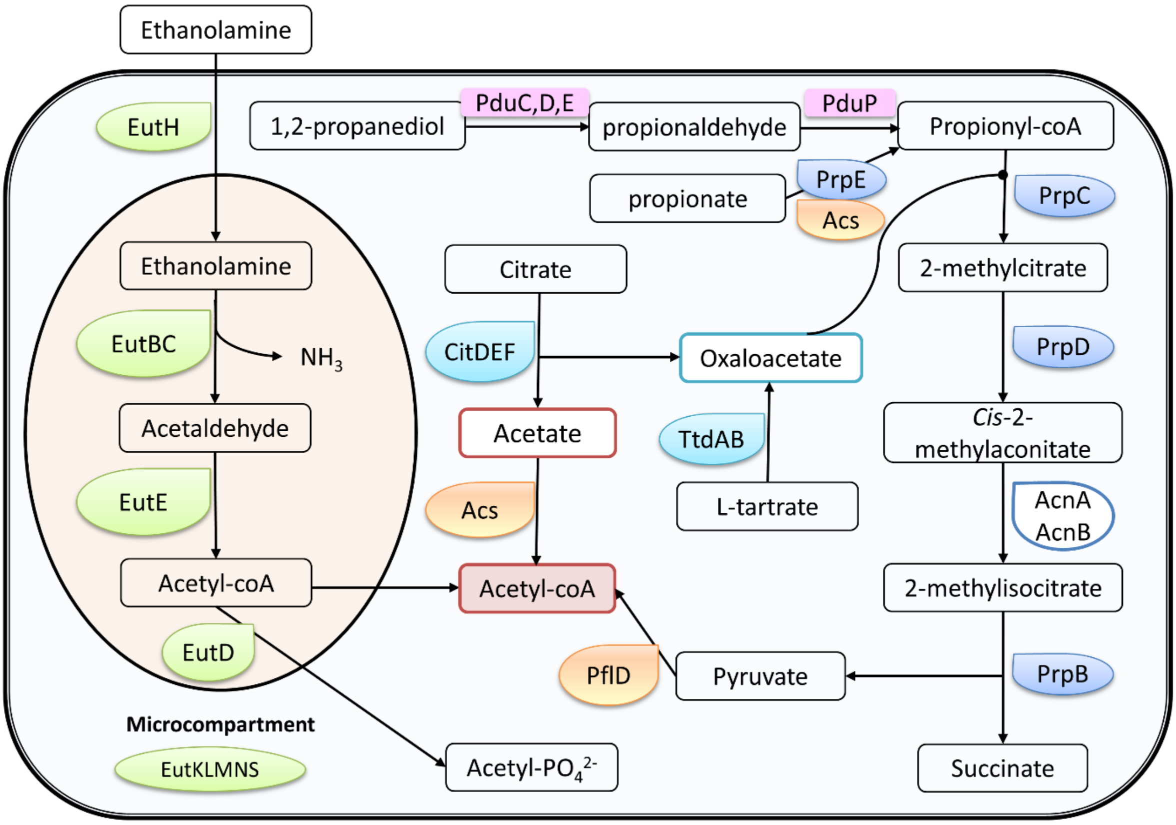

Figure S6: Schematic showing the interplay between ethanolamine degradation, citrate and L-tartrate fermentation and the 2-methylcitrate pathway under bile conditions.

Ethanolamine is metabolized by AIEC to produce nitrogen and acetyl-CoA. Citrate is converted to acetate and oxaloacetate by citrate lyase. Oxaloacetate, which is necessary for the methylcitrate pathway, is also supplied for L-tartrate fermentation and amino acid degradation. Propionyl-coA from various degradation products is metabolized to produce pyruvate, which can be converted to acetyl-coA. The genes that exhibited an increase in mRNA concentrations in bacteria grown with bile salts in comparison to without bile salts encoded proteins involved in ethanolamine degradation (green boxes), 2-methylcitrate pathway (blueberry boxes), citrate and L-tartrate fermentation (cyan boxes) and 1,2-propandediol degradation (pink boxes).

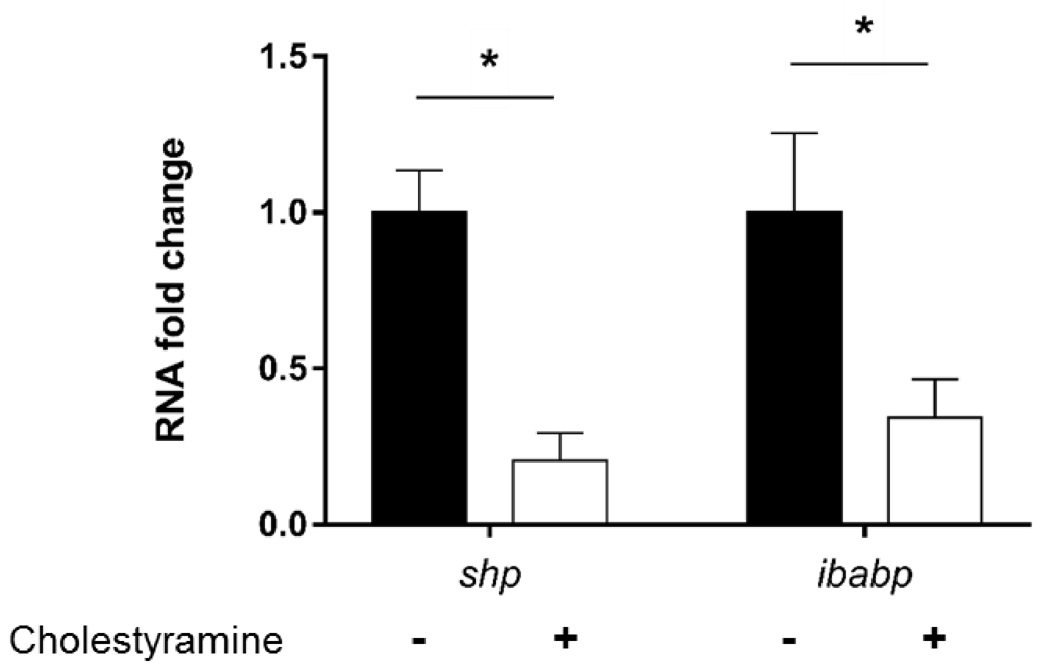

Figure S7: Effect of cholestyramine on mRNA levels of *shp* and *ibabp* in the ileum of mice. Cholestyramine is a bile-acid sequestrant that binds bile acids in the intestine to prevent their reabsorption. As a result, this decreases the feedback suppression of hepatic bile-acid synthesis from cholesterol. Values are means ± SEM. Statistical analysis was performed with a Mann-Whitney test; * p<0.05.

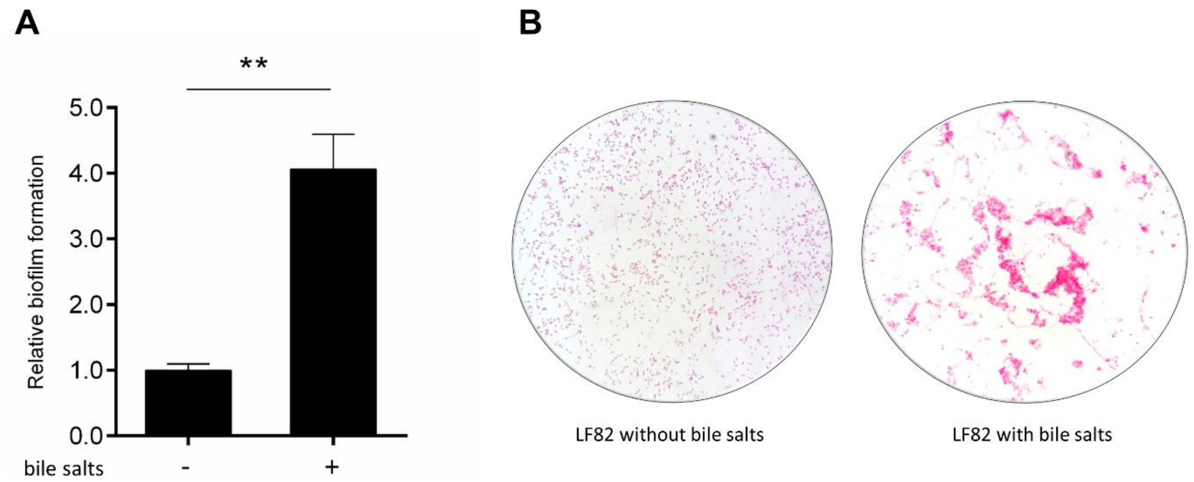

Figure S8: Bile salts induce biofilm formation and autoaggregation of bacteria.

(**A**) Crystal violet quantification of biofilm formation. Strains were grown in mM9-EA medium supplemented or not with 1% bile salts. After 4 h and 30 min of incubation at 37°C, surface-adherent biofilm formation was measured by staining. Values are means ± SD of at least three independent experiments. (**B**) Autoaggregation of bacteria from visualized by microscopy after Gram staining (1000x magnification). Statistical analysis was performed with a Mann-Whitney test; **, $p < 0.01$.

Table S1: qRT-PCR validation of selected genes

Gene number	Gene name	Product name	qRT-PCR fold change (Log_2)	RNA-seq fold change (Log_2)
LF82_0581	*eutB*	ethanolamine ammonia-lyase heavy chain	1.882	2.55
LF82_1739	*prpB*	Methylisocitrate lyase	2.59	4.561
LF82_0729	*folX*	D-erythro-7.8-dihydroneopterin triphosphate epimerase	-0.802	-0.262
LF82_1619	*pfkA*	6-phosphofructokinase isozyme 1	-2.43	-1.725
LF82_0286	*cfa*	Cyclopropane-fatty-acyl-phospholipid synthase	-2.70	-2.70
LF82_p715	LF82_p715	Ornithine carbamoyltransferase chain I	1.383	1.99

Table S2: Variations of the expression of genes involved in biofilm formation

name	product	gene size (bp)	RNA-seq values (minimal medium) RPKM	RNA-seq values (bile salts) RPKM	Fold change	Fold change (Log2)	p value	type of virulence gene
acrE	acriflavine resistance protein E	1157	619	127	**4.9**	**2.29**	1.181E-15	biofilm
acrF	acriflavine resistance protein F	3104	1355	665	**2.0**	**1.03**	2.395E-05	biofilm
bcsA	Cellulose synthase catalytic subunit [UDP-forming]	2618	2103	1498	1.4	0.49	5.361E-02	biofilm
bcsB	Cyclic di-GMP-binding protein	2339	993	612	1.6	0.70	2.248E-03	biofilm
bcsC	Cellulose synthase operon protein C	3473	1450	978	1.5	0.57	1.407E-02	biofilm
bcsZ	endoglucanase	1112	693	519	1.3	0.42	6.640E-02	biofilm
cpsB	Mannose-1-phosphate guanylyltransferase [GDP]	1436	448	83	**5.4**	**2.44**	4.227E-15	biofilm
cpsG	Phosphomannomutase (colanic acid biosynthesis)	1370	160	25	**6.3**	**2.65**	2.972E-14	biofilm
csgA	Major curlin subunit	455	74	206	**2.8**	**1.48**	4.550E-06	pili
csgB	Minor curlin subunit	455	67	138	**2.1**	**1.05**	8.281E-04	pili
fcl	GDP-L-fucose synthetase	965	163	41	**4.0**	**1.99**	6.025E-09	biofilm
fimA	Type-1 fimbrial protein. A chain	548	7094	21306	**3.0**	**1.59**	1.812E-06	pili
fimB	Type 1 fimbriae regulatory protein fimB	602	413	428	1.0	0.05	8.898E-01	pili
fimC	chaperone protein fimC	725	2840	3333	1.2	0.23	4.971E-01	pili
fimD	Outer membrane usher protein fimD	2636	3624	6328	1.7	0.80	9.777E-03	pili
fimE	Type 1 fimbriae regulatory protein fimE	596	809	450	**-1.8**	**-0.84**	1.584E-04	pili
fimF	Protein fimF	530	206	415	**2.0**	**1.008**	1.064E-04	pili
fimG	Protein fimG	503	208	528	**2.5**	**1.34**	4.303E-08	pili
fimH	Protein fimH	902	574	920	1.6	0.681	5.124E-03	pili
fimI	Fimbrin-like protein fimI	539	3699	5088	1.4	0.46	2.335E-01	pili
flhC	flagellar transcriptional activator flhC	578	1026	2437	**-2.4**	**-1.249**	2.296E-05	pili
flhD	Transcriptional activator flhD	350	1331	5144	**-3.9**	**-1.95**	6.999E-08	pili

name	product	gene size (bp)	RNA-seq values (minimal medium) RPKM	RNA-seq values (bile salts) RPKM	Fold change	Fold change (Log2)	p value	type of virulence gene
gmd	GDP-mannose 4.6-dehydratase	1121	321	67	**4.8**	**2.256**	7.842E-12	biofilm
ppdD	Prepilin peptidase-dependent protein D	440	116	128	1.1	0.14	6.745E-01	pili
tnaA	Tryptophanase	1430	2970	545	**5.5**	**2.45**	8.555E-15	other
tnaB	Low affinity tryptophan permease	1247	1573	558	**2.8**	**1.495**	4.406E-11	other
wcaA	colanic acid biosynthesis glycosyl transferase	839	264	52	**5.1**	**2.34**	1.053E-11	biofilm
wcaB	colanic acid biosynthesis acetyltransferase	488	109	22	**4.9**	**2.305**	5.687E-10	biofilm
wcac	colanic acid biosynthesis glycosyl transferase	1217	405	63	**6.4**	**2.684**	2.750E-16	biofilm
wcad	colanic acid polymerase	1217	656	150	**4.4**	**2.13**	6.018E-13	biofilm
wcae	colanic acid biosynthesis glycosyl transferase	746	275	60	**4.6**	**2.202**	5.112E-12	biofilm
wcaF	colanic acid biosynthesis acetyltransferase	548	116	26	**4.4**	**2.14**	8.126E-09	biofilm
wcaI	colanic acid biosynthesis glycosyl transferase	1214	241	25	**9.8**	**3.29**	7.506E-18	biofilm
wcaJ	colanic biosynthesis UDP-glucose lipid carrier	1394	461	65	**7.0**	**2.81**	1.056E-17	biofilm
wcaK	colanic acid biosynthesis protein wcaK	1280	244	58	**4.2**	**2.08**	3.060E-10	biofilm
wcal	colanic acid biosynthesis glycosyltransferase	1220	449	564	-1.3	-0.33	1.687E-01	biofilm
wcaM	colanic acid biosynthesis protein wcaM	1394	826	546	1.5	0.598	1.219E-02	biofilm
wza	polysaccharide export protein wza	1139	349	74	**4.7**	**2.226**	5.062E-13	biofilm
wzb	Low molecular weight protein-tyrosine-phosphatase	443	107	20	**5.4**	**2.434**	9.992E-08	biofilm
wzc	Tyrosine-protein kinase wzc	2162	650	139	**4.7**	**2.23**	9.343E-17	biofilm
yadC	fimbrial-like protein yadC	1262	1607	1611	1.0	0.004	9.912E-01	pili
yadK	orf	596	103	271	2.6	1.392	1.260E-01	pili
yadL	orf	608	92	160	1.8	0.81	3.809E-03	pili
ydeP	Protein ydeP	2279	568	862	1.5	0.601	1.532E-02	pili
ydeQ	fimbrial-like protein ydeQ	914	113	250	**2.2**	**1.148**	5.470E-04	pili

name	product	gene size (bp)	RNA-seq values (minimal medium) RPKM	RNA-seq values (bile salts) RPKM	Fold change	Fold change (Log2)	p value	type of virulence gene
ydeR	fimbrial-like protein ydeR	503	339	421	1.2	0.315	2.302E-01	pili
ydes	fimbrial-like protein ydes	530	181	323	1.8	0.83	7.688E-03	pili
yehA	orf	1034	351	465	1.3	0.41	1.382E-01	pili
yehB	outer membrane usher protein yehB	2480	243	822	**3.4**	**1.761**	2.152E-11	pili
yehC	fimbrial chaperone yehC	674	27	223	**8.1**	**3.02**	7.172E-18	pili
yehD	orf	542	28	124	**4.4**	**2.14**	5.677E-11	pili
yehE	orf	281	438	452	1.0	0.04	8.737E-01	pili
LF82_p366	orf	857	683	608	-1.1	-0.169	4.454E-01	pili
LF82_p367	fimbrial-like protein yfcP	446	40	149	**3.7**	**1.89**	1.704E-08	pili
LF82_p368	Yfc fimbriae subunit YfcQ	479	39	99	**2.5**	**1.33**	1.296E-04	pili
LF82_p369	orf	503	28	47	1.7	0.74	8.409E-02	pili
LF82_p370	periplasmic chaperone YfcS	752	100	261	**2.6**	**1.38**	5.212E-06	pili
LF82_p371	outer membrane usher YfcU	2654	980	1178	1.2	0.27	3.152E-01	pili
LF82_p372	Yfc fimbriae subunit YfcV	566	429	143	**-3.0**	**-1.58**	2.635E-08	pili
LF82_p461	orf	983	140	680	**4.8**	**2.273**	8.694E-21	PAI-IV/group 2 capsul
LF82_p462	Polysialic acid capsule synthesis protein KpsE	1148	187	629	**3.4**	**1.75**	1.511E-15	PAI-IV/group 2 capsul
LF82_p463	KpsD protein	1676	260	802	**3.1**	**1.625**	2.746E-14	PAI-IV/group 2 capsul
LF82_p464	3-deoxy-manno-octulosonatecytidylyltransferase	740	54	296	**5.4**	**2.443**	1.120E-17	PAI-IV/group 2 capsul
LF82_p465	KpsC protein	2027	324	1002	**3.1**	**1.627**	4.229E-14	PAI-IV/group 2 capsul
LF82_p466	KpsS protein	1259	246	916	**3.7**	**1.90**	4.083E-18	PAI-IV/group 2 capsul
LF82_p467	Transposase within prophage	1124	212	547	**2.6**	**1.37**	2.025E-08	PAI-IV/group 2 capsul
LF82_p468	orf	983	222	544	**2.4**	**1.29**	5.044E-06	PAI-IV/group 2 capsul
LF82_p469	orf	1166	333	773	**2.3**	**1.22**	8.374E-07	PAI-IV/group 2 capsul

name	product	gene size (bp)	RNA-seq values (minimal medium) RPKM	RNA-seq values (bile salts) RPKM	Fold change	Fold change (Log2)	p value	type of virulence gene
LF82_p470	orf	1190	430	709	**1.6**	**0.72**	6.277E-04	PAI-IV/group 2 capsul
LF82_p471	orf	1250	212	476	**2.3**	**1.17**	8.775E-04	PAI-IV/group 2 capsul
LF82_p472	glycerol-3-phosphate cytidyltransferase	395	137	490	**3.6**	**1.842**	5.837E-14	PAI-IV/group 2 capsul
LF82_p473	KpsT protein	674	119	280	**2.4**	**1.233**	1.382E-05	PAI-IV/group 2 capsul
LF82_p474	KpsM protein	776	250	498	**2.0**	**1.00**	6.626E-05	PAI-IV/group 2 capsul
LF82_p509	Yqi fimbriae subunit YgiL	563	129	206	1.6	0.69	1.835E-02	pili
LF82_p510	outer membrane usher protein YqiG	2522	1748	1134	-1.5	-0.62	8.810E-03	pili
LF82_p511	fimbrial chaperone yqiH	749	779	620	-1.3	-0.33	1.175E-01	pili
LF82_p512	Yqi fimbrial adhesin	1049	3757	1483	-2.5	**-1.34**	1.502E-06	pili
LF82_p527	fimbrial adhesin (*auf*)	1130	190	558	**2.9**	**1.552**	4.169E-09	pili
LF82_p528	fimbrial chaperone (*auf*)	749	116	377	**3.3**	**1.71**	1.416E-09	pili
LF82_p529	minor fimbrial subunit precursor (*auf*)	515	129	277	**2.1**	**1.103**	1.024E-04	pili
LF82_p530	minor fimbrial subunit precursor (*auf*)	563	187	481	**2.6**	**1.361**	4.641E-07	pili
LF82_p531	Outer membrane usher protein AufC	2594	422	1586	**3.8**	**1.91**	1.131E-11	pili
LF82_p532	Auf fimbiral chaperone 1	752	324	487	1.5	0.589	1.275E-02	pili
LF82_p533		704	882	621	-1.4	-0.51	2.276E-02	pili
LF82_p548	major fimbrial subunit	524	180	294	1.6	0.71	1.020E-02	pili
LF82_p549	fimbriae	1052	1495	1122	-1.3	-0.414	1.111E-01	pili
LF82_p550	Outer membrane usher protein lpfC precursor	2531	1297	1444	1.1	0.154	5.427E-01	pili
LF82_p551	Fimbrial chaperone protein	686	96	193	2.0	1.00	1.393E-03	pili
LF82_p552	fimbrial-like protein	524	244	188	-1.3	-0.37	1.943E-01	pili

Table S3: Bacterial strains and plasmids used in this study

Strain or plasmid	Relevant characteristics	Reference
LF82	AIEC reference strain	[9]
LF82Δ*eutB*	LF82 isogenic mutant deleted for the *eutB* gene	this study
LF82Δ*eutE*	LF82 isogenic mutant deleted for the *eutE* gene	this study
LF82Δ*eutB*/p*eutB*	LF82Δ*eutB* containing plasmid pBK-CMV carrying *eutB*	this study
LF82Δ*citF*	LF82 isogenic mutant deleted for the *citF* gene	this study
MG1655	*Escherichia coli* K-12 OR:H48:K-	Laboratory stock
BW25141	Plasmid pKD4 carrying a kanamycin resistance cassette	[10]
pKOBEG	pBAD cloning vector harboring a λ phage redγβα operon; chloramphenicol resistant	[11]
pCP20	Ampicillin and chloramphenicol resistant plasmid that shows temperature-sensitive replication and thermal induction of FLP synthesis	[10]

Table S4: Primers used in this study

Primer	Sequence (5'→3')	PCR product size	Use
d-eutB-F	GTGCGGTGACGGTGAAATCACTCGCATTTCCTTCCTGAGG GAACGACTTGTAGGCTGGAGCTGCTTC	-	construction of isogenic mutant
d-eutB-R	TACAATTTCTTCAATCTGTTTTTGATCCATGATGTGTTATC TCCGCGTCACATATGAATATCCTCCTTAG		
d-eutE-F	GAGGTGGTGTCTGGCGGTCAGGTCATTTTCCACAAATAA GGCAGAACATCGTAGGCTGGAGCTGCTTC	-	construction of isogenic mutant
d-eutE-R	TTGCAGACGTGGAGTGAGCCATTGTTCATCGTGCGCCATC GGTTACTCCTCATATGAATATCCTCCTTAG		
d-citF-F	TTATTCCTTCACCTGATGCACAACATCGATCACCGAGCCA TCGCGGTAACGTAGGCTGGAGCTGCTTCG	-	construction of isogenic mutant
d-citF-R	ATGACGCAGAAAATTGAACAATCTCAACGACAAGAACG GGTAGCGGCCTGCATATGAATATCCTCCTTAG		
eutBEcoR1-F	GGAATTCATGAAACTAAAGACCAC	1.362 bp	eutB cloning
eutBBamH1-R	CGGGATCCCGGGATCCTCAGAAGAACA		
eutB-F	AGTATCGCCGCGCAAATCTA	151 bp	RT-PCR amplification
eutB-R	CCCTGGGTCGGAATGTTGAA		
eutC-F	GCAGGTCGTTATTTCTGATG	228 bp	RT-PCR amplification
eutC-R	GCTTTCTGACTGCCCCAACC		
eutD-F	GTTTTTCGGATGCGTTAGA	198 bp	RT-PCR amplification
eutD-R	CCAGCGATGAGCAAATTCTT		
eutE-F	TGAAACTGACCGCAGAGCAG	206 bp	RT-PCR amplification
eutE-R	TCAGTTCGGTCACGGCAAAC		
eutG-F	GCTGGTTTCTGACATCTCTG	213 bp	RT-PCR amplification
eutG-R	CCTGGCCGTTAAAGGCATC		
eutH-F	CTGGTGGTCTGGTAGCGAT	206 bp	RT-PCR amplification
eutH-R	CAAGACCGAGGGTGATCAAT		
eutL-F	GCGTACCGGTTCTTATCTCT	191 bp	RT-PCR amplification
eutL-R	TGGCTACCGGTTAAAAATGC		

Primer	Sequence (5’→3’)	PCR product size	Use
citF-F<br>citF-R	CGTGCCGGGTTAACAGCGAT<br>GGTACTGAGCGCGCTGGAAA	248 bp	RT-PCR amplification
prpB-F<br>prpB-R	TGGATGCGAAAACCGATCCT<br>TGGCGAGTTCGGTAATTGCT	149 bp	RT-PCR amplification
folX-F<br>folX-R	CCACTACCCCGCCGATAAAG<br>TAGCATACGTCACCCAGTG	174 bp	RT-PCR amplification
pfkA-F<br>pfkA -R	TCGTGGCGATTACCGAACAT<br>CAGCAGCAGATCGATAGCGT	178 bp	RT-PCR amplification
cfa-F<br>cfa -R	TTTGCGGTGGTGGATCGTAA<br>TCGCTGGACTGAGCAATCTG	157 bp	RT-PCR amplification
LF_715-F<br>LF_715-R	GGGAGAGCCAAAAGAAGCCT<br>CAGTCCTTTGAGGCCGTAGG	182 bp	RT-PCR amplification
tufA-F<br>tufa-R	GACATGGTGATGACGAAGA<br>GCTCTGGTTCCGGAATGTA	199 bp	RT-PCR amplification
16S-F<br>16S-R	ATGACCAGCCACACTGGAAC<br>CTTCCTCCCCGCTGAAAGTA	150 bp	RT-PCR amplification
Shp-F	CGATCCTCTTCAACCCAGATG	-	RT-PCR amplification
Shp-R	AGGGCTCCAAGACTTCACACA	-	RT-PCR amplification
IBABP-F	CAGGAGACGTGATTGAAAGGG	-	RT-PCR amplification
IBABP-R	GCCCCCAGAGTAAGACTGGG	-	RT-PCR amplification

References

1. Darfeuille-Michaud, A. Adherent-invasive Escherichia coli: a putative new E. coli pathotype associated with Crohn’s disease. *Int. J. Med. Microbiol. IJMM* **292,** 185–193 (2002).
2. Darfeuille-Michaud, A. *et al.* Presence of adherent Escherichia coli strains in ileal mucosa of patients with Crohn’s disease. *Gastroenterology* **115,** 1405–1413 (1998).
3. Miquel, S. *et al.* Complete Genome Sequence of Crohn’s Disease-Associated Adherent-Invasive E. coli Strain LF82. *PLoS ONE* **5,** e12714 (2010).
4. Karp, P. D. *et al.* The EcoCyc Database. *EcoSal Plus* **6,** (2014).

5. Krzywinski, M. *et al.* Circos: An information aesthetic for comparative genomics. *Genome Res.* **19,** 1639–1645 (2009).
6. Keseler, I. M. *et al.* EcoCyc: fusing model organism databases with systems biology. *Nucleic Acids Res.* **41,** D605–D612 (2013).
7. O'Toole, G. A. & Kolter, R. Initiation of biofilm formation in Pseudomonas fluorescens WCS365 proceeds via multiple, convergent signalling pathways: a genetic analysis. *Mol. Microbiol.* **28,** 449–461 (1998).
8. Hennequin, C., Aumeran, C., Robin, F., Traore, O. & Forestier, C. Antibiotic resistance and plasmid transfer capacity in biofilm formed with a CTX-M-15-producing Klebsiella pneumoniae isolate. *J. Antimicrob. Chemother.* **67,** 2123–2130 (2012).
9. Barnich, N. & Darfeuille-Michaud, A. Adherent-invasive Escherichia coli and Crohn's disease. *Curr. Opin. Gastroenterol.* **23,** 16–20 (2007).
10. Datsenko, K. A. & Wanner, B. L. One-step inactivation of chromosomal genes in Escherichia coli K-12 using PCR products. *Proc. Natl. Acad. Sci. U. S. A.* **97,** 6640–6645 (2000).
11. Chaveroche, M. K., Ghigo, J. M. & d'Enfert, C. A rapid method for efficient gene replacement in the filamentous fungus Aspergillus nidulans. *Nucleic Acids Res.* **28,** E97 (2000).
